# Integrative analysis of immunogenic PANoptosis and experimental validation of cinobufagin-induced activation to enhance glioma immunotherapy

**DOI:** 10.1186/s13046-025-03301-1

**Published:** 2025-02-03

**Authors:** Yonghua Cai, Heng Xiao, Shuaishuai Xue, Peng Li, Zhengming Zhan, Jie Lin, Zibin Song, Jun Liu, Wei Xu, Qixiong Zhou, Songtao Qi, Xi’an Zhang, Ziyi Luo

**Affiliations:** 1 Department of Neurosurgery, Institute of Brain Diseases, Nanfang Hospital, Southern Medical University, Guangzhou, Guangdong 510515 China; 2 Department of Neurosurgery, Guangzhou Women and Children’s Medical Center, Guangdong Provincial Clinical Research Center for Child Health, Guangzhou Medical University, Guangzhou, 510623 China; 3 Neurosurgery Center, Department of Functional Neurosurgery, The Neurosurgery Institute of Guangdong Province, Zhujiang Hospital, Southern Medical University, Guangzhou, Guangdong China; 4 Department of Neurosurgery, the 2nd affiliated hospital, Jiangxi Medical College, Nanchang University, Nanchang, Jiangxi 330006 China

**Keywords:** Glioma, PANoptosis, Immunotherapy, Cinobufagin, Tumor immune microenvironment

## Abstract

**Background:**

Glioma, particularly glioblastoma (GBM), is a highly aggressive tumor with limited responsiveness to immunotherapy. PANoptosis, a form of programmed cell death merging pyroptosis, apoptosis, and necroptosis, plays an important role in reshaping the tumor microenvironment (TME) and enhancing immunotherapy effectiveness. This study investigates PANoptosis dynamics in glioma and explores the therapeutic potential of its activation, particularly through natural compounds such as cinobufagin.

**Methods:**

We comprehensively analyzed PANoptosis-related genes (PANoRGs) in multiple glioma cohorts, identifying different PANoptosis patterns and constructing the PANoptosis enrichment score (PANoScore) to evaluate its relationship with patient prognosis and immune activity. Cinobufagin, identified as a PANoptosis activator, was evaluated for its ability to induce PANoptosis and enhance anti-tumor immune responses both in vitro and in vivo GBM models.

**Results:**

Our findings indicate that high PANoScore gliomas showed increased immune cell infiltration, particularly effector T cells, and enhanced sensitivity to immunotherapies. Cinobufagin effectively induced PANoptosis, leading to increased immunogenic cell death, facilitated tumor-associated microglia/macrophages (TAMs) polarization towards an M1-like phenotype while augmenting CD4+/CD8 + T cell infiltration and activation. Importantly, cinobufagin combined with anti-PD-1 therapy exhibited significant synergistic effects and prolonged survival in GBM models.

**Conclusions:**

These findings highlight the therapeutic potential of PANoptosis-targeting agents, such as cinobufagin, in combination with immunotherapy, offering a promising approach to convert “cold” tumors into “hot” ones and improving glioma treatment outcomes.

**Supplementary Information:**

The online version contains supplementary material available at 10.1186/s13046-025-03301-1.

## Introduction

Gliomas, which originate from glial cells in the central nervous system, represent a diverse class of primary brain tumors with significant heterogeneity in histopathology and biological behavior. High-grade gliomas, especially grade IV glioblastoma multiforme (GBM), exhibit aggressive proliferation and resistance to standard therapies, contributing to poor patient outcomes [1, 2]. Immunotherapy has emerged as a promising therapeutic strategy; however, its success in GBM is hampered by the “cold” immunosuppressive microenvironment of the tumor. This tumor microenvironment (TME), characterized by limited T cell infiltration and a predominance of immunosuppressive tumor-associated microglia/macrophages (TAMs), presents a substantial barrier to effective immune activation [3–5], highlighting the need for therapies that can reshape the TME to increase immune responsiveness.

PANoptosis, a novel form of programmed cell death (PCD), integrates pyroptosis, apoptosis, and necroptosis through a unique multiprotein scaffold called the PANoptosome. Specific triggers initiate PANoptosome assembly, leading to the activation of downstream PCD executors: caspase-3/7 (CASP3/7)-mediated apoptosis, gasdermin-D (GSDMD)-mediated pyroptosis, and mixed lineage kinase domain-like protein (p-MLKL)-mediated necroptosis [6–12]. This unified cell death pathway is thought to play an essential role in regulating immune responses within the tumor microenvironment. PANoptosis induces cancer cell death and simultaneously recruits and activates immune cells, amplifying the antitumor response and underscoring its therapeutic potential, particularly in highly immune-evasive cancers [13–16]. This is especially pertinent for GBM, where immunosuppressive TAMs and limited cytotoxic T cell infiltration create substantial obstacles to effective immunotherapy. Despite the promising role of PANoptosis in modulating cell death and immune responses, its specific implications in gliomas, including GBM, remain insufficiently explored. Initial research has begun to unravel the dysregulation of PANoptosis-related pathways and their contribution to glioma aggressiveness and resistance to therapy [17–19]. However, comprehensive investigations examining the interactions of PANoptosis within the glioma microenvironment and its therapeutic implications are still lacking.

Cinobufagin, a bioactive compound derived from the skin secretions of Venenum bufonis, has been used in traditional Chinese medicine for centuries due to its analgesic and anticancer properties [20, 21]. Recent studies have highlighted cinobufagin's potential as an adjunct in cancer immunotherapy, with evidence showing that it can induce cell death in cancer cells while enhancing immune responses against tumors. Notably, cinobufagin appears to activate dendritic cells and T cells, thus bolstering antitumor immunity [22, 23]. Additionally, research suggests that cinobufagin may sensitize tumors to immune checkpoint inhibitors, making it a promising candidate for amplifying the effectiveness of existing immunotherapies [24]. Although interest in cinobufagin as an antitumor agent has grown, its effects on gliomas, particularly GBM, remain under-investigated. Preliminary studies have shown that cinobufagin can inhibit glioma cell proliferation and induce apoptosis [25], yet detailed research on its mechanisms of action, especially concerning PANoptosis and its influence on the immune microenvironment in gliomas, is needed. 

**Fig. 1:**
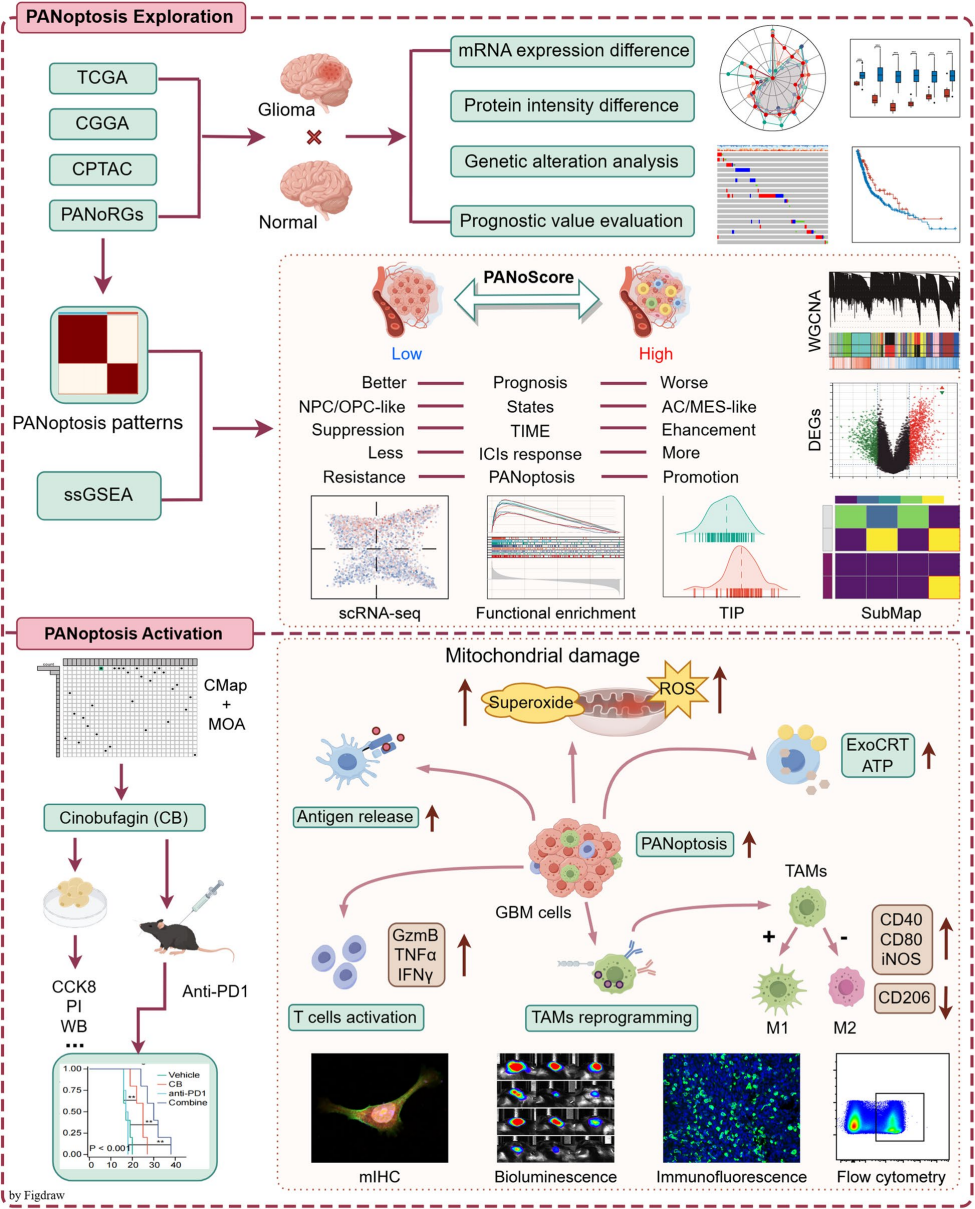
Flow chart of this study. The figure illustrates the experimental design and flow of this article. (Abbreviations: TCGA: The Cancer Genome Atlas; CGGA: Chinese Glioma Genome Atlas; CPTAC: Clinical Proteomic Tumor Analysis Consortium; PANoRGs: PANoptosis-Related Genes; ssGSEA: Single-sample Gene Set Enrichment Analysis; TIME: Tumor Immune Microenvironment; TIP: Tracking Tumor Immunophenotype; ICIs: Immune checkpoint inhibitors; NPC-like: Neural Progenitor-like; OPC-like: Oligodendrocyte Precursor Cell; AC-like: Astrocyte-like; MES-like: Mesenchymal-like; scRNA-seq: Single-cell RNA sequencing; WGCNA: Weighted correlation network analysis; DEGs: Differentially expressed genes; SubMap: Subclass mapping; CMap: Connectivity Map; MOA: Mode-of-action; CB: Cinobufagin; CCK8: Cell Counting Kit-8; PI: Propidium iodide; WB: Western blot; ROS: Reactive oxygen species; ExoCRT: Externalization of calreticulin to the cell membrane; TAMs: Tumor-associated microglia/macrophages; mIHC: Multiplex immunohistochemistry)

In summary, the complex challenges presented by GBM underscore the need for innovative treatments that address not only tumor cell survival but also the underlying immunosuppressive environment. Our comprehensive investigation of PANoptosis and the utilization of natural compounds such as cinobufagin offers exciting potential to enhance current therapies and improve patient outcomes in this formidable disease. The experimental design is detailed in Fig. 1.

## Materials and methods

### Data preparation

The bulk RNA sequencing data were obtained from The Cancer Genome Atlas (TCGA, https://portal.gdc.cancer.gov/), the Chinese Glioma Genome Atlas (CGGA, http://www.cgga.org.cn/index.jsp) [26], and the Clinical Proteomic Tumor Analysis Consortium (CPTAC, https://proteomic.datacommons.cancer.gov/pdc/) [27]. These datasets comprised four cohorts: “TCGA-Glioma” (*n* = 639), “CGGA-Glioma” (*n* = 688), “TCGA-GBM-IDHwt” (*n* = 150), and “CPTAC-GBM-IDHwt” (*n* = 93). Additionally, single-cell RNA sequencing (scRNA-seq) data for IDH wild-type GBM (GBM-IDHwt) were sourced from Neftel et al. (GSE131928) [28]. The TCGA-Glioma cohort was utilized to explore the inter-tumor heterogeneity and clinical relevance of PANoptosis in gliomas, whereas the CGGA-Glioma cohort served for validation. The TCGA-GBM-IDHwt cohort was used to examine the relationships between PANoptosis and aggressiveness, as well as tumor microenvironment (TME) characteristics, with corroboration from the CPTAC-GBM-IDHwt cohort. The scRNA-seq data primarily facilitated the investigation of the intratumor heterogeneity of PANoptosis and its regulators in GBM.

###  Bioinformatics analysis and machine learning

Consensus clustering [29] combined with principal component analysis (PCA), was performed to identify and verify distinct PANoptosis patterns. Single-sample Gene Set Enrichment Analysis (ssGSEA) [30] was used to calculate the PANoptosis enrichment score (PANoScore). Functional analyses were conducted via Gene Ontology (GO) [31], Kyoto Encyclopedia of Genes and Genomes (KEGG) [32], and Gene Set Enrichment Analysis (GSEA) [33]. Weighted correlation network analysis (WGCNA) [34] and differentially expressed gene (DEG) analysis [35] were used to identify genes associated with the PANoScore. To evaluate the TME and antitumor immunity, we utilized multiple tools, including Tracking Tumor Immunophenotype (TIP) [36], ImmunophenScore (IPS) [37], ESTIMATE [38], TIMER [39], MCPCounter [40], EPIC [41], xCell [42], CIBERSORT [43], and quanTIseq [44]. Subclass mapping (SubMap) [45] and Tumor Immune Dysfunction and Exclusion (TIDE) [46] analyses assessed the implication of PANoScore on immunotherapy response and efficacy. Drug sensitivity was assessed via the Genomics of Drug Sensitivity in Cancer (GDSC) database [47], whereas the Connectivity Map (CMap) database, with mode-of-action (MOA) analysis [48], aided in identifying small-molecule compounds that could enhance PANoptosis activation in tumors. We obtained scRNA-seq data and associated metadata, including cell-type annotations, from the study by Neftel et al. (GSE131928) [28], which provides a comprehensive view of GBM’s transcriptional landscape with detailed annotations for distinct cellular states: astrocyte-like (AC-like), mesenchymal-like (MES-like), neural-progenitor-like (NPC-like), and oligodendrocyte-progenitor-like (OPC-like). To examine the relationship between PANoptosis and cellular states, we calculated the PANoScore for each cell via ssGSEA and compared the PANoScores across these four states.

### Cell lines and cell culture

Primary GBM cells (GBM0603, GBM0108, and GBM0109, referred to as G001, G003, and G007 in this study) were obtained from Zeng, Kunlin et al. [49]. GBM cell lines (A172, U87, and T98G) were purchased from the American Type Culture Collection (ATCC). The cells were cultured in DMEM supplemented with 10% fetal bovine serum (FSP500, ExCell) and 1% penicillin‒streptomycin (15140-122, Gibco) at 37 °C in a humidified incubator with 5% CO₂. The cells were passaged with 0.25% trypsin-EDTA solution (25200072, Gibco) at 80–90% confluence.

### Cell Counting Kit-8 (CCK-8)

GBM cell lines (A172, U87, and T98G) and primary GBM cells (G001, G003, and G007) were seeded in 96-well plates at a density of 3000 cells per well. After 24 h of treatment with cinobufagin, 10 µL of CCK-8 reagent (C0040, Beyotime) was added to each well, followed by a 2-hour incubation at 37 °C. The absorbance was then measured at 450 nm via a microplate reader.

### Propidium iodide (PI) staining

G003 and G007 cells (500,000 per well) were seeded in 6-well plates and treated with cinobufagin for 24 h. The cells were stained with PI (C2015M, Beyotime), diluted 1000-fold, and incubated at 37 °C for 30 min in the dark. PI-positive cells, indicating cell death, were analyzed via flow cytometry following the manufacturer’s instructions.

### Western blot (WB) analysis

After 24 h of treatment with cinobufagin, tumor cell proteins were extracted via RIPA buffer containing protease and phosphatase inhibitors. Protein concentrations were measured with a BCA protein assay kit (P0009, Beyotime). Equal amounts of protein (30–50 µg) were separated on SDS-PAGE gels and transferred onto PVDF membranes (IPVH00010, Millipore). The membranes were blocked with 5% non-fat milk in TBST for 1 h at room temperature. The membranes were incubated overnight at 4 °C with primary antibodies against the following proteins: CASP3 (9662, Cell Signaling Technology), c-CASP3 (9662, Cell Signaling Technology), CASP7 (9491, Cell Signaling Technology), c-CASP7 (9492, Cell Signaling Technology), GSDMD (66387-1-lg, Proteintech), GSDME (ab215191, Abcam), p-MLKL (18640, Cell Signaling Technology), and MLKL (14993, Cell Signaling Technology). After being washed with TBST, the membranes were incubated with HRP-conjugated secondary antibodies for 1 h at room temperature. The protein bands were visualized via an enhanced chemiluminescence reagent (P0018FM, Beyotime). The chemiluminescence signals were captured via the ChemiScope-S6-SE imaging system. After the target protein bands were detected, the membranes were stripped and re-probed with β-actin (4967, Cell Signaling Technology) as a loading control. Band intensities were quantified using ImageJ software for further analysis.

### Reactive oxygen species (ROS) production detection

For intracellular ROS, the cells were incubated with 10 µM DCFH-DA (S0033, Beyotime) in serum-free medium at 37 °C for 30 min in the dark. After washing, DCF fluorescence was measured via flow cytometry. To detect mitochondrial ROS, the cells were incubated with 5 µM MitoSOX Red (S0061, Beyotime) in HBSS at 37 °C for 10 min in the dark. After washing, MitoSOX Red fluorescence Red was analyzed by flow cytometry following the manufacturer’s instructions.

### Measurement of mitochondrial mass

After 24 h of cinobufagin treatment, the cells were stained with MitoTracker Orange CMTMRos (M7510, Invitrogen) at a final concentration of 100 nM in serum-free medium and incubated at 37 °C for 30 min in the dark. After washing, MitoTracker fluorescence was measured by flow cytometry following the manufacturer’s instructions.

### Detection of immunogenic cell death (ICD) hallmarks

After 24 h of treatment with cinobufagin, ICD markers were assessed via two approaches. To detect the externalization of calreticulin to the cell membrane (ExoCRT), the cells were stained with a calreticulin antibody (NBP1-47518APC, Novus) and incubated for 30 min in the dark. After washing, ExoCRT fluorescence was analyzed via flow cytometry following the manufacturer’s protocol. To measure ATP release in the cell supernatant, an ADP/ATP ratio assay kit (MAK135-1KT, Sigma-Aldrich) was used. The supernatant was collected, and ATP levels were quantified via a luminescence microplate reader following the kit instructions.

### Mouse models and therapeutic trials

To establish intracranial glioma mouse models, 500,000 GL261 or CT2A cells suspended in 4 µL of PBS were injected into the brains of 4–8-week-old C57BL/6J mice purchased from the Guangdong Medical Laboratory Animal Center. The procedure was performed using a 10 µL Hamilton microsyringe for precise delivery. The mice were anesthetized, and the injection site was located 2 mm to the right of the bregma and 0.5–1 mm posterior. The needle was inserted to a depth of 4 mm and then retracted by 1 mm before injection. The cells were delivered at a constant rate over 4 min, followed by a 2-minute pause to prevent reflux before slowly withdrawing the needle. The mice were monitored closely during recovery to ensure their well-being and minimize stress. For in vivo therapeutic trials, the mice were randomly assigned to four groups: vehicle control, cinobufagin treatment, anti-PD1 monoclonal antibody treatment, and cinobufagin combined with anti-PD1 monoclonal antibody treatment. Cinobufagin (HY-N0421, MedChemExpress) was administered intraperitoneally at 5 mg/kg, starting on day 6 post-implantation, every other day for a total of five doses. Anti-PD1 monoclonal antibody (BP0273, BioXcell) was administered intraperitoneally at 10 µg/g, starting on day 7 post-implantation, every two days for a total of three doses. Treatment regimens aimed at evaluating the effects of individual and combination therapies on tumor progression.

### Bioluminescent monitoring

On day 15 post-implantation, bioluminescence imaging was conducted to track tumor growth. The mice were anesthetized with isoflurane (Rayward Life Technologies Inc., Shenzhen, China) and administered D-Luciferin potassium salt (15 mg/kg, D&B, Shanghai, China) via intraperitoneal injection. Imaging was performed 10 min postinjection via the AniView Kirin IVIS Lumina system (Boluteng Biotechnology, Guangzhou, China) to assess the tumor burden.

### Harvesting of mouse tissues

On day 16 post-implantation, the mice were anesthetized and transcardially perfused with 100 mL of saline followed by 100 mL of 4% paraformaldehyde (PFA) via the left ventricle. The brain, heart, liver, spleen, lungs, and kidneys were carefully collected. All harvested tissues were postfixed in 4% PFA at 4 °C for 48 h, then dehydrated, embedded in paraffin, and processed into tissue blocks for further analysis.

### Toxicology analysis experiments

Body weight was monitored throughout the treatment period to assess systemic toxicity. Serum was collected at the end of treatment for a Comprehensive Chemistry Test. The heart, liver, spleen, lungs, and kidneys were harvested and subjected to hematoxylin and eosin (HE) staining for histopathological evaluation.

### Immunofluorescence (IF) and multiplex immunohistochemistry (mIHC)

 For IF staining of CD45 (ab317446, Abcam), CD4 (67786-1-IG, Proteintech), CD8a (ab217344, Abcam), IBA1 (ab178846, Abcam), GZMB (ab255598, Abcam), and CD206 (24595, Cell Signaling Technology), thin Sect. (1 μm) were prepared via a microtome and mounted on glass slides. The sections were dewaxed in xylene, rehydrated through a graded alcohol series, and treated with 3% H2O2 for 10 min to block endogenous peroxidase activity. Antigen retrieval was performed by microwaving in 2% EDTA buffer (ZLI-9069, ZSGB-BIO). The sections were then blocked with 3% BSA for 1 h at room temperature, followed by overnight incubation at 4 °C with primary antibodies. After washing, the sections were incubated with secondary antibodies (anti-rabbit Alexa Fluor 488, A11008, Invitrogen; anti-mouse Alexa Fluor 488, A11001, Invitrogen) for 1 h in the dark. Nuclei were counterstained with DAPI (ab104139, Abcam), and images were captured via a Zeiss LSM980 confocal microscope (20×). For mIHC of phosphorylated MLKL (AF7420, Affinity), cleaved N-terminal GSDME (55879, Cell Signaling Technology), and cleaved CASP3 (9662, Cell Signaling Technology), the Tyramide Signal Amplification (TSA) method was used with the TSA-RM-275 kit (10001100, Panovue). Thin sections were dewaxed, rehydrated, and subjected to antigen retrieval as described. After blocking, the sections were incubated sequentially with primary antibodies against phosphorylated MLKL, cleaved N-terminal GSDME, and cleaved CASP3, each followed by incubation with the corresponding HRP-conjugated secondary antibodies. TSA amplification was conducted via PPD550 for phosphorylated MLKL, PPD570 for cleaved N-terminal GSDME, and PPD650 for cleaved CASP3, following the manufacturer’s instructions. Signal stripping was performed between each round of staining to prevent cross-reactivity. Finally, the sections were counterstained with DAPI, and images were captured via a Zeiss LSM980 confocal microscope (60×). Data analysis and image processing were performed using ImageJ software.

### Flow cytometry analysis

Brain tumors or splenocytes were dissected, minced, and digested with 5 mg/ml collagenase II (17101-015, Gibco) and 0.1 mg/ml recombinant DNase I (D8071, Solarbio) at 37 °C for 30 min. The cell suspensions were then passed through a 70 μm filter to ensure a single-cell suspension. Red blood cells were removed using ACK lysis buffer (abs9101, Absin). The remaining cells were resuspended in staining buffer (554656, BD Biosciences) and blocked with anti-CD16/CD32 antibodies (553141, BD Biosciences) at 4 °C for 15 min. For cell surface staining, the cells were incubated with the following antibodies on ice for 45 min: anti-CD45 (557659, BD Biosciences), anti-CD4 (abs182366, Absin), anti-CD8a (551162, BD Biosciences), anti-Ki67 (563756, BD Biosciences), anti-CD69 (104539, Biolegend), anti-PD-1 (109110, Biolegend), anti-PD-L1 (741014, BD Biosciences), anti-F4/80 (565411, BD Biosciences), anti-CD80 (740130, BD Biosciences), anti-CD40 (124622, Biolegend), anti-MHC class I (12–5999-82, Invitrogen), and anti-MHC class II (107614, Biolegend) antibodies. After surface marker staining, Fixable Viability Stain 620 (564996, BD Biosciences) was applied to assess cell viability, followed by fixation and permeabilization using fixation/permeabilization buffer (88–8824-00, eBioscience) for intracellular staining. After permeabilization, the cells were incubated with the following antibodies at 4 °C for 50 min: TNFα (506333, Biolegend), IFNγ (505842, Biolegend), GzmB (396414, Biolegend), CD206 (141723, Biolegend), and iNOS (53–5920-82, Invitrogen). Flow cytometry was conducted via the CytoFLEX system, and the data were analyzed with FlowJo software (v.10).

### Statistical analysis and visualization

Statistical analyses and data visualization were performed via GraphPad Prism 9 and R software (version 4.2.1). The data are presented as the means ± SEMs unless otherwise specified in the figure legends. Kaplan-Meier survival curves were generated, and statistical comparisons were made using the log-rank test. For comparisons between two groups, an unpaired t-test (equal variances) or Welch’s t-test (unequal variances) was used. For experiments involving more than two groups, one-way ANOVA (equal variances) or Welch’s ANOVA (unequal variances) was performed unless otherwise stated. Statistical significance was indicated as *P < 0.05, **P < 0.01, and ***P < 0.001.

## Results

### Molecular landscape of PANoptosis regulators revealed via multi-omics

**Fig. 2:**
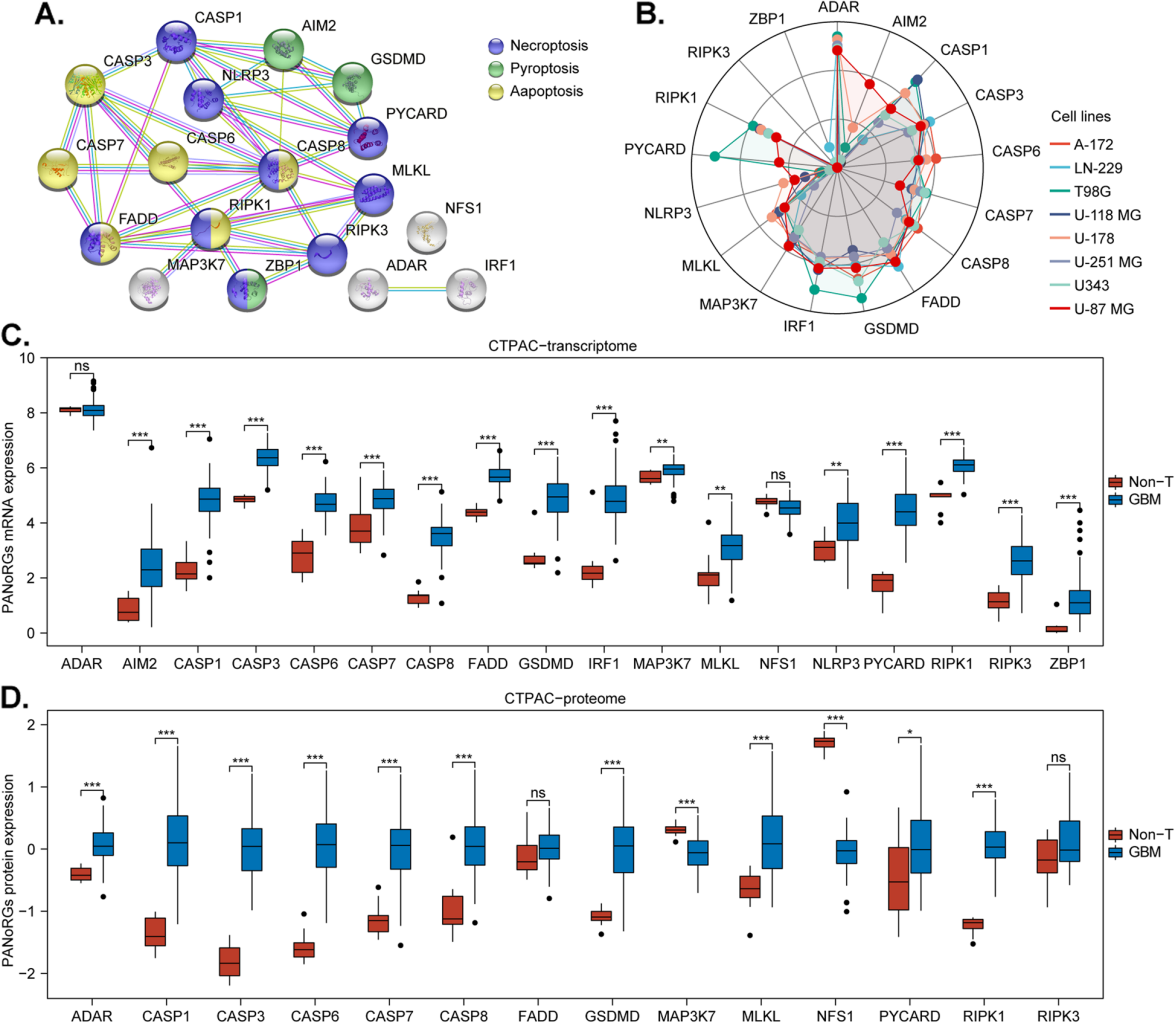
Molecular landscape of PANoptosis regulators revealed by multi-omics. A Protein‒protein interaction (PPI) network of PANoRGs. **B **Radar chart illustrating the expression distribution of PANoRGs across various glioma cell lines. **C **Comparison of mRNA expression between GBM and nontumor brain tissues. **D **Protein intensity comparison of PANoRGs between GBM and nontumor tissues. **E **Genomic alteration frequencies of PANoRGs in glioma samples. **F **Differences in the top 10 genes with the highest alteration event frequencies between the altered and unaltered PANoRGs groups in glioma. **G **Kaplan-Meier survival curves comparing overall survival (OS) between the altered and unaltered PANoRGs groups

We collected 18 PANoRGs and categorized them into four categories (driver, suppressor, PANoptosome component, and executor) (Table S1) according to previous studies [6–14, 50–60]. We then analyzed their transcriptomic, proteomic, genomic, and epigenomic characteristics, as well as their prognostic value in glioma. The protein-protein interaction (PPI) network demonstrated that PANoRGs exhibit intimate and reciprocal interactions (Fig. 2A), highlighting extensive crosstalk among pyroptosis, apoptosis, and necroptosis. RNA-seq data from the CCLE indicated that over 80% of PANoRGs were expressed in the majority of the cell lines (Fig. 2B). Comparisons of mRNA expression within the CPTAC-Glioma cohort revealed that 89% of PANoRGs (16/18) were more highly expressed in GBM than in non-tumor brain tissues (Fig. 2C). Additionally, we explored the mRNA expression of PANoRGs across various clinical and molecular phenotypes—including grade, histology, IDH mutation status, 1p19q codeletion, and MGMT promoter methylation—in the TCGA-Glioma cohort (Supplement Fig. 1) and CGGA-Glioma cohort (Supplement Fig. 2), revealing noticeable heterogeneity in the transcriptional levels of most PANoRGs across glioma samples. Subsequently, proteomic analysis of the CPTAC-Glioma cohort revealed that 71% of PANoRGs (10/14; note: data for AIM2, IRF1, NLRP3, and ZBP1 were not obtained) are more highly expressed in GBM, whereas 14% (2/14, MAP3K7 and NFS1) are expressed at lower levels in GBM tissues than in non-tumor brain tissues (Fig. 2D). Furthermore, genomic analysis displayed that PANoRGs were altered in 99 (12%) of 794 glioma samples, with no individual alteration frequency exceeding 3% (Supplement Fig. 3A). Among the top 10 genes with the highest alteration event frequencies, 90% (9/10) exhibited significantly different alteration frequencies between the altered and unaltered PANoRGs groups (Supplement Fig. 3B). Survival analysis revealed that, compared with the altered group, the unaltered group had decreased survival (Supplement Fig. 3C). Additionally, DNA methylation analysis demonstrated that the promoter methylation levels of AIM2, CASP1, CASP8, NLRP3, and ZBP1 are significantly lower in gliomas than in non-tumor brain tissues (Supplement Fig. 3D-U). Finally, univariate Cox analysis revealed that 78% of the PANoRGs (14/18, hazard ratio > 1 and P value < 0.05) were negatively correlated with overall survival (OS) in both the TCGA and CGGA glioma cohorts (Table S2). In conclusion, the diverse expression profiles and significant alterations of PANoRGs in glioma underscore their critical involvement in the regulation of PANoptosis and their potential impact on progression and prognosis.

**Fig. 3:**
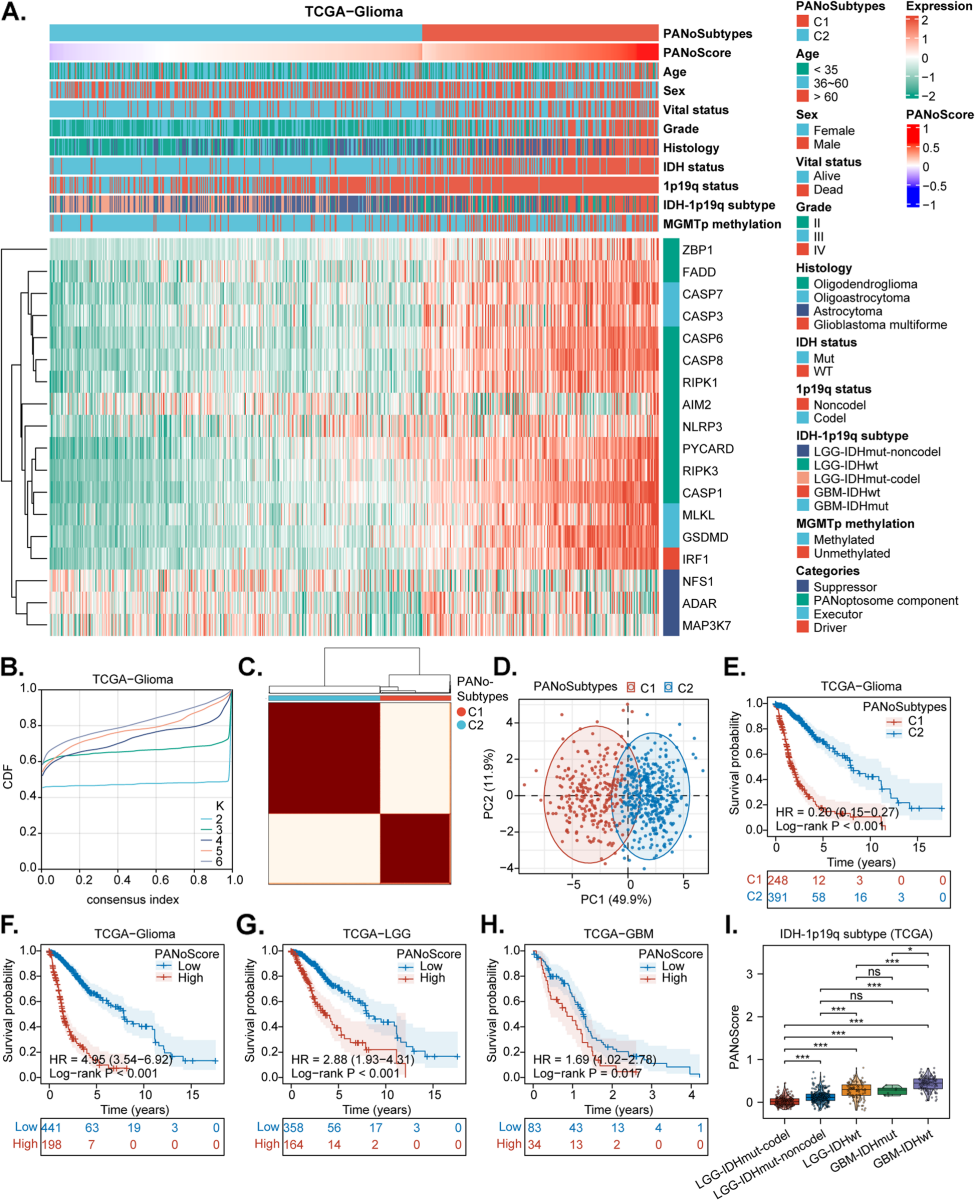
Clinical Characteristics and Prognostic Value of PANoptosis Patterns. **A **Expression of PANoRGs and distribution of PANoScores stratified by different clinical and molecular phenotypes in the TCGA-Glioma cohort. **B **Empirical cumulative distribution function (CDF) plot determining the optimal number of clusters (K = 2). **C **Clustering analysis identifying two distinct PANoptosis patterns (C1 and C2). **D **Principal component analysis (PCA) plot illustrating the separation between C1 and C2. **E **Survival analysis comparing C1 and C2. **F-H **Kaplan-Meier survival curves comparing OS between the high and low PANoScore groups across gliomas, including low-grade glioma (LGG) and glioblastoma multiforme (GBM). **I **Distribution of the PANoScore among various molecular entities in the TCGA-Glioma cohort

### Clinical characteristics and prognostic value of PANoptosis patterns

To assess the status of PANoptosis in glioma samples, we performed cluster analysis to identify distinct PANoptosis patterns on the basis of PANoRGs expression. The empirical cumulative distribution function plot (Fig. 3B) indicated that the optimal cluster number was two (K = 2), resulting in the classification of samples in the TCGA-Glioma cohort into two PANoptosis patterns: C1 and C2 (Fig. 3C). Notably, the two PANoptosis patterns were well separated by principal component analysis (PCA), confirming the cluster analysis results (Fig. 3D). Survival analysis revealed that patients in C1 had significantly shorter survival than those in C2 (Fig. 3E), indicating the existence of two distinct PANoptosis statuses that are associated with the clinical prognosis in glioma. To further quantify the enrichment of PANoptosis, we calculated the PANoScore via ssGSEA based on PANoRGs. Our findings indicated that high expression of drivers (IRF1), executors (GSDMD, CASP7, and MLKL), and most PANoptosome components (FADD, RIPK1, CASP6, CASP8, CASP1, PYCARD, and RIPK3) was correlated with a high PANoScore. In contrast, high expression of suppressors (NFS1, ADAR, and MAP3K7) was associated with a lower PANoScore (Fig. 3A). This finding suggests that greater potential for PANoptosis activation occurs with a higher PANoScore in gliomas. Meanwhile, the PANoScore in C1 was significantly higher than that in C2 **(**Fig. 3A and Supplement Fig. 4A**)**, suggesting that C1 is more likely to exhibit the potential for PANoptosis activation in gliomas. Survival analysis demonstrated that a higher PANoScore was associated with a poorer prognosis (Fig. 3F-H). Univariate and multivariate Cox analyses revealed that the PANoScore serves as an independent prognostic predictor linked to adverse outcomes in glioma patients (Table S3). Additionally, we found that the PANoScore was significantly associated with various clinical factors, including age, grade, IDH mutation status, 1p19q codeletion status, MGMT promoter methylation status, and histology (Fig. 3A and Supplement Fig. 4B-K). Based on IDH mutation status, 1p/19q codeletion status, and conventional histopathology, gliomas can be classified into five molecular entities: (I) LGG with IDH mutation and 1p/19q noncodeletion (LGG-IDHmut-noncodel); (II) LGG with IDH mutation and 1p/19q codeletion (LGG-IDHmut-codel); (III) LGG with wild-type IDH (LGG-IDHwt); (IV) GBM with IDH mutation (GBM-IDHmut); and (V) GBM with wild-type IDH (GBM-IDHwt) [61–63]. Our analysis demonstrated that the PANoScore was significantly higher in the IDHwt counterparts than in the IDHmut counterparts, with the greatest enrichment observed in the GBM-IDHwt counterparts (Fig. 3I). Parallel analyses were conducted in the CGGA-Glioma cohort, yielding similar results (SupplementFigs. 5 and 6).

**Fig. 4:**
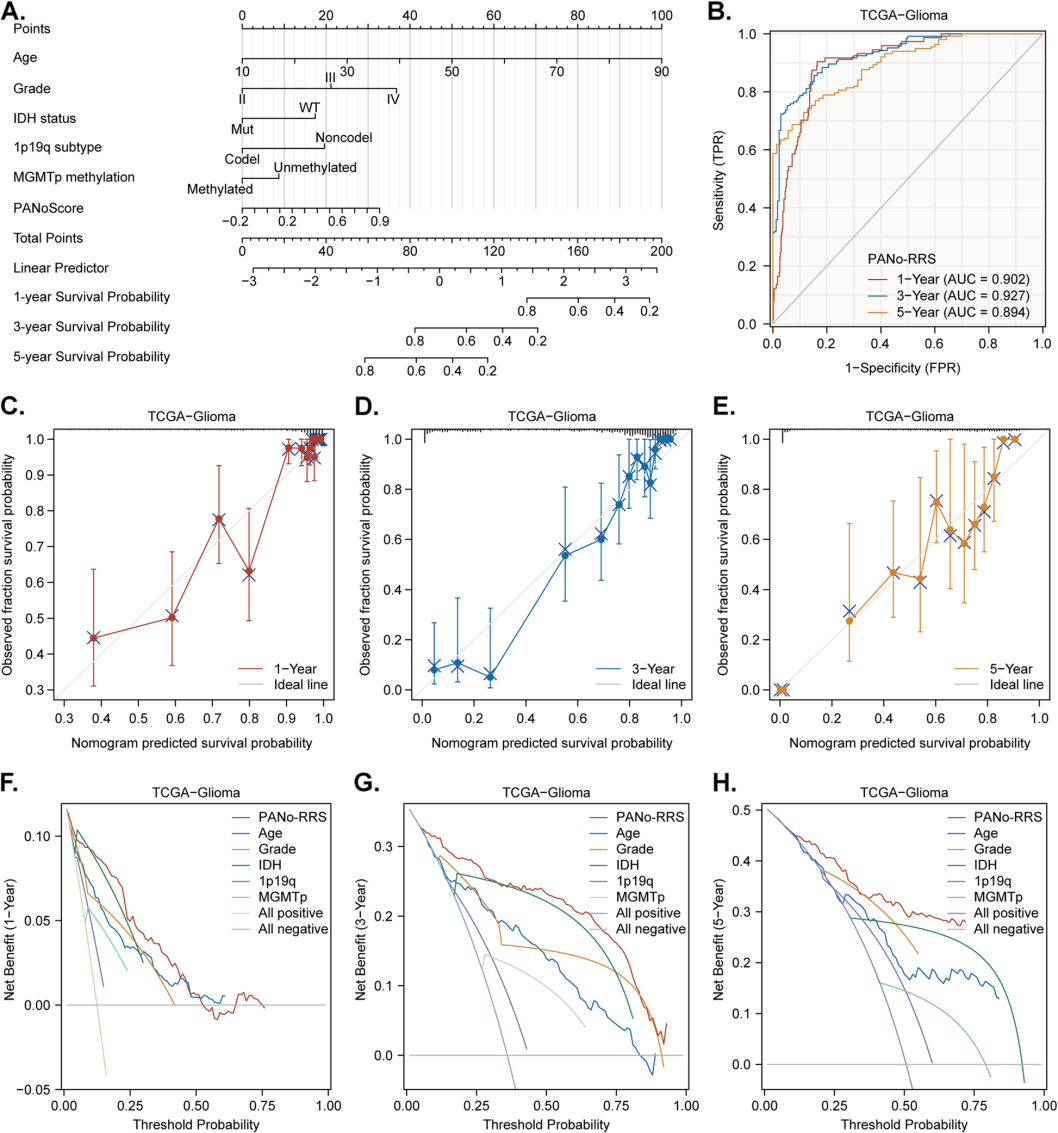
Development and validation of the PANoptosis-related risk score (PANo-RRS) for prognostic prediction.** A **Nomogram illustrating the integration of the PANoScore with clinical and molecular features for predicting outcomes. **B **Receiver operating characteristic (ROC) curves demonstrating the accuracy of the PANo-RRS for predicting 1-, 3-, and 5-year OS. **C-E** Calibration curves assessing the prediction consistency of PANo-RRS. **F-H **Decision curve analysis (DCA) showing the clinical utility of PANo-RRS

**Fig. 5:**
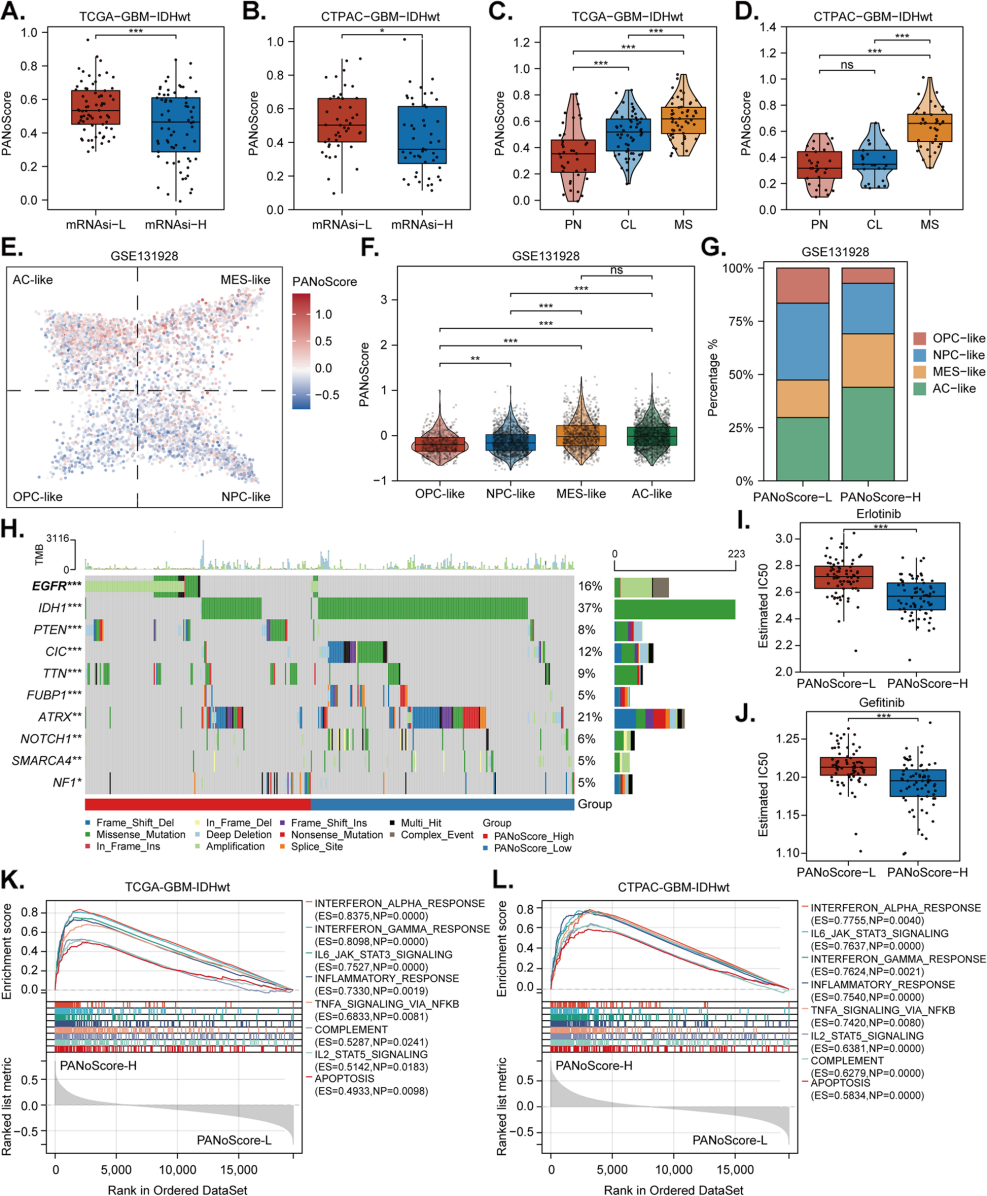
Linking PANoptosis Enrichment to Cellular Neoplastic Heterogeneity and Aggressiveness.** A-B** Comparison of PANoScore between the low and high mRNA expression-based stemness index (mRNAsi) groups in GBM. **C-D **Distribution of PANoScore among different transcriptional subtypes: proneural (PN), classical (CL), and mesenchymal (MS). **E **Single-cell RNA sequencing (scRNA-seq) analysis showing the PANoScore distribution across astrocyte-like (AC-like), mesenchymal-like (MES-like), neural-progenitor-like (NPC-like), and oligodendrocyte-progenitor-like (OPC-like) cellular states. **F **Comparison of the PANoScore among AC-like, MES-like, NPC-like, and OPC-like cells. **G **Proportion of AC-like, MES-like, NPC-like, and OPC-like cells in high and low PANoScore groups. **H **Oncoplot displaying mutational differences between the high and low PANoScore groups. **I-J **Estimated half maximal inhibitory concentration (IC50) of EGFR-targeted drugs (gefitinib and erlotinib) compared between the low and high PANoScore groups. **K-L **Gene set enrichment analysis (GSEA) showing enrichment of immune-related pathways in high-PANoScore GBM samples

**Fig. 6:**
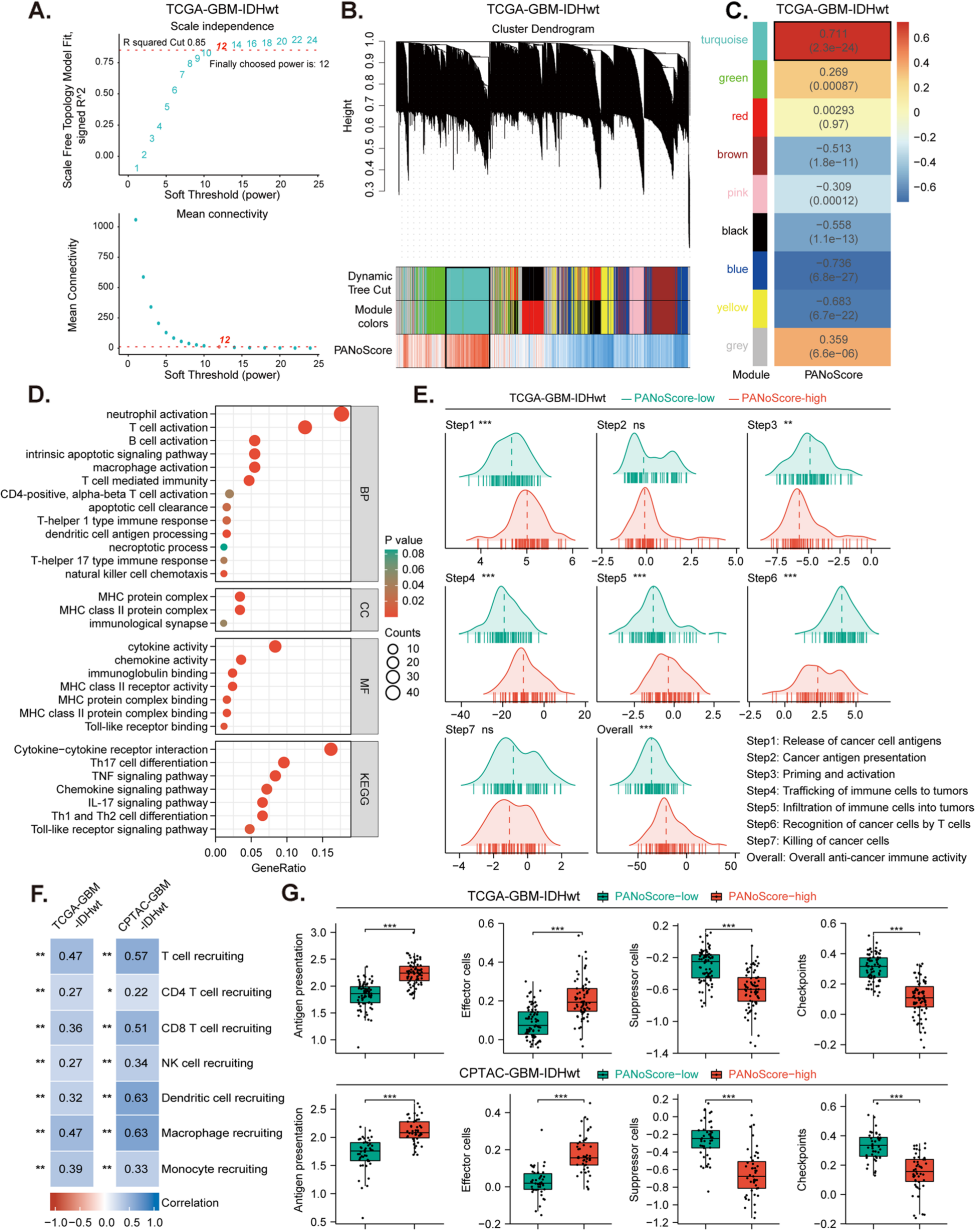
Interconnection between PANoptosis enrichment and tumor immune dynamics.** A **Selection of soft threshold power (R² > 0.85), with power 12 chosen for optimal fit using Weighted Gene Co-expression Network Analysis (WGCNA) in the TCGA-GBM-IDHwt cohort. **B **Cluster dendrogram with a dynamic tree cut showing gene modules correlated with PANoScore. **C **Correlation heatmap between gene coexpression modules and the PANoScore. **D **Gene Ontology (GO) and Kyoto Encyclopedia of Genes and Genomes (KEGG) enrichment analyses of common genes from turquoise modules. **E **Tracking Tumor Immunophenotype (TIP) analysis comparing immune activity across seven steps of the cancer immunity cycle between the high and low PANoScore groups. **F **Correlation between the PANoScore and immune cell recruitment. **G **Immunophenoscore (IPS) analysis comparing immunophenotypes between the high and low PANoScore groups

Despite the strong associations between several factors—such as IDH mutation status, 1p19q codeletion, and MGMT promoter methylation—and survival in glioma, accurate prognostic markers remain elusive. To fill this gap, we integrated the PANoScore with multiple clinical factors, including tumor grade, IDH status, 1p19q status, and the MGMT promoter status, and developed a PANoptosis-related risk score (PANo-RRS) via multivariate Cox analysis in the TCGA-Glioma cohort. The detailed calculations for the PANo-RRS are presented in Table S4 and visualized as a nomogram (Fig. 4A). We subsequently performed receiver operating characteristic (ROC) curve and calibration analyses to evaluate the prognostic accuracy of the PANo-RRS for OS prediction and decision curve analysis (DCA) to assess its clinical utility. The ROC curve (Fig. 4B) and calibration curves (Fig. 4C-E) demonstrated that the PANo-RRS can accurately predict the OS of glioma patients at 1, 3, and 5 years, whereas the DCA curves (Fig. 4F-H) indicated that the PANo-RRS provides the maximum net benefit. Additionally, survival analysis revealed that the PANo-RRS maintains a high degree of predictive stability across various clinical and molecular subgroups of gliomas, except for the GBM-IDHmut subgroup (Supplement Fig. 7). Finally, we validated the predictive accuracy, stability, and clinical utility of the PANo-RRS in the CGGA-Glioma cohort, which yielded consistent results (Supplement Fig. 8). These results suggest that the PANo-RRS, which combines PANoScore with clinical factors, is a highly promising prognostic prediction index for glioma.

**Fig. 7:**
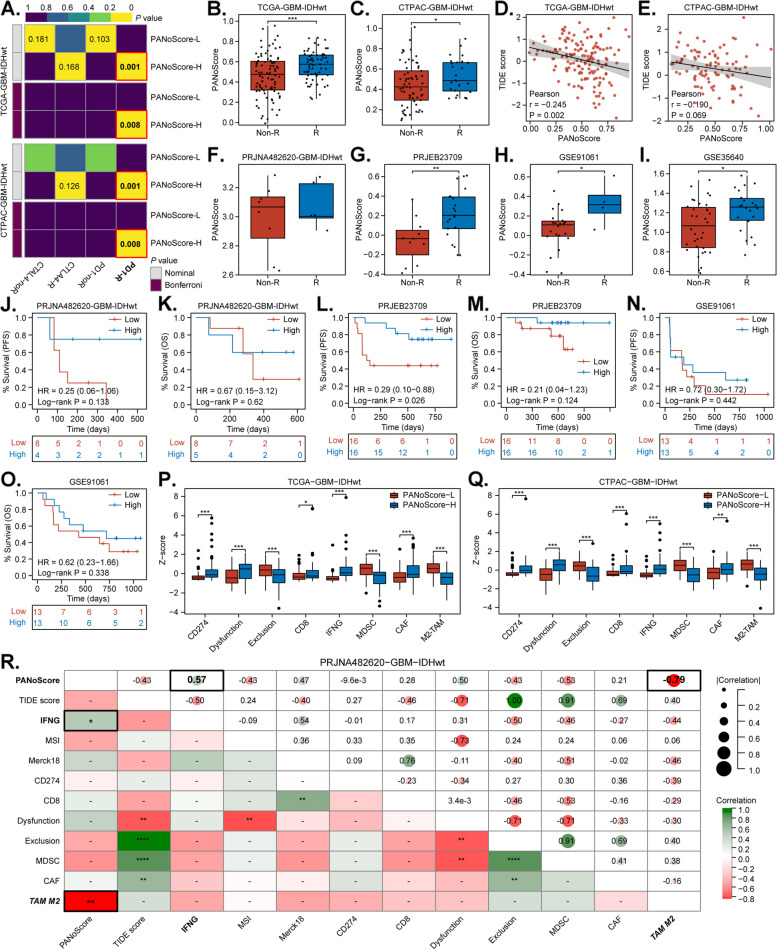
Evaluating enhanced PANoptosis in GBM immunotherapy.** A **Subclass Mapping (SubMap) analysis predicting response to anti-PD1/CTLA4 therapies based on PANoScore in the TCGA-GBM-IDHwt and CPTAC-GBM-IDHwt cohorts. **B-C **Comparison of the PANoScore between responders (R) and nonresponders (non-R) to immunotherapy as identified by Tumor Immune Dysfunction and Exclusion (TIDE) analysis. **D-E **Correlations between the PANoScore and TIDE score. **F-I **PANoScore comparisons between R and non-R patients across clinical immunotherapy cohorts (PRJNA482620, PRJEB23709, GSE91061, and GSE35640). **J-O **Kaplan-Meier curves showing the associations between the PANoScore and progression-free survival (PFS) and OS in immunotherapy cohorts. **P-Q **TIDE analysis comparing immune characteristics, including T cell dysfunction and exclusion, immune checkpoints, cytokines, myeloid-derived suppressor cells (MDSCs), M2-type tumor-associated macrophages (M2-TAMs), and cancer-associated fibroblasts (CAFs), between the high and low PANoScore groups in the TCGA-GBM-IDHwt and CPTAC-GBM-IDHwt cohorts. **R **Correlation matrix illustrating the relationships between the PANoScore, T cell dysfunction and exclusion, immune checkpoints, cytokines, MDSCs, M2-TAMs, and CAFs in the GBM immunotherapy cohort (PRJNA482620)

**Fig. 8:**
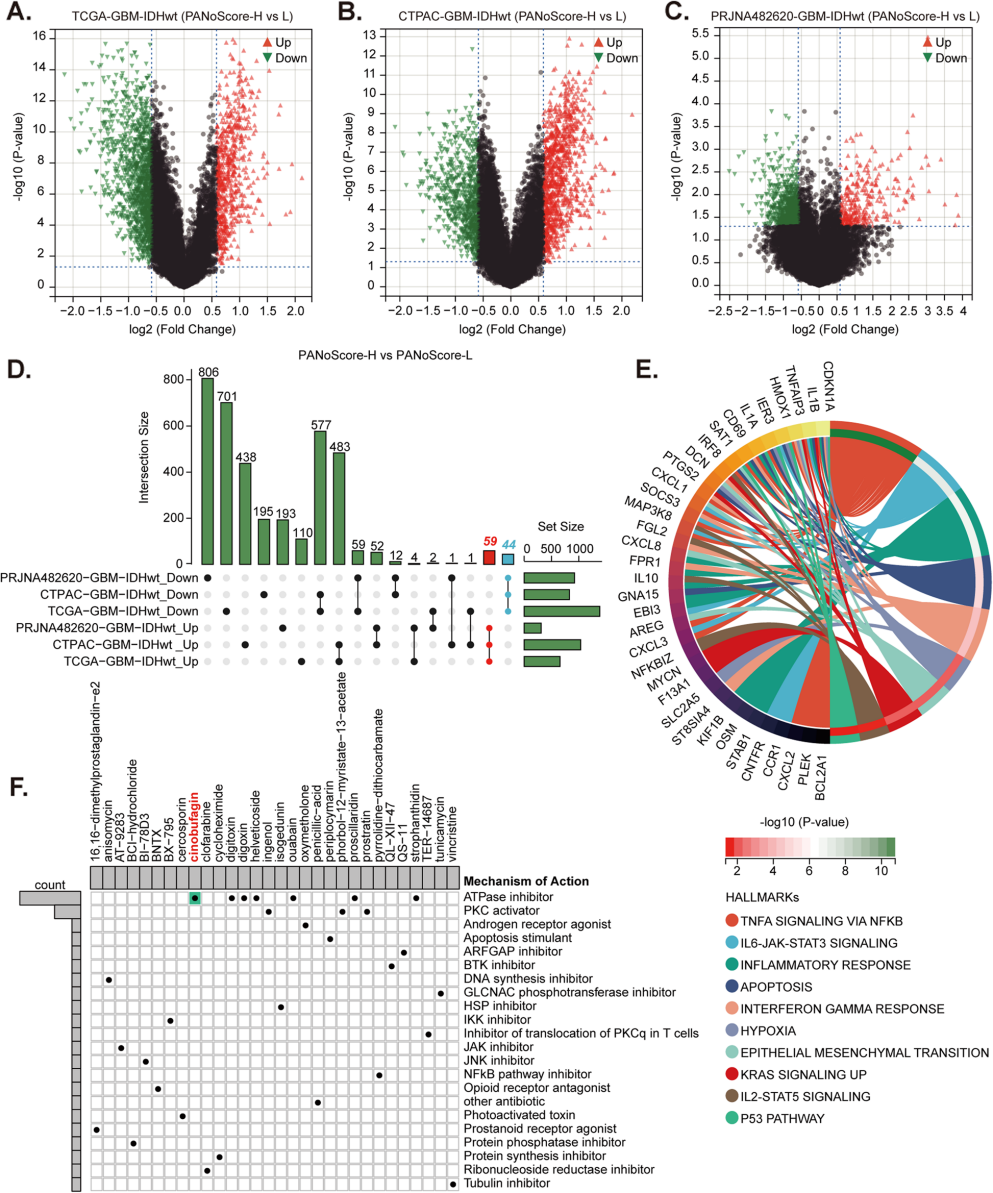
Identifying potential small-molecule activators of PANoptosis in GBM.** A-C **Differential expression gene (DEG) volcano plots showing DEGs between the high and low PANoScore groups in the TCGA-GBM-IDHwt, CPTAC-GBM-IDHwt, and PRJNA482620-GBM-IDHwt cohorts. **D **UpSet plot illustrating common DEGs across the three cohorts. **E **Chord diagram visualizing the functional enrichment analysis of common DEGs. **F **Candidate small-molecule compounds and their mechanisms of action predicted through the Connectivity Map (CMap) database and Mechanism of Action (MOA) analysis

### Linking PANoptosis enrichment to cellular neoplastic heterogeneity and aggressiveness

Considering the high enrichment of PANoptosis in GBM-IDHwt, we next focused on investigating its biological influences in this context. GBM contains cellular hierarchies with self-renewing, highly tumorigenic stem cells (glioblastoma stem cells, GSCs) at the apex, contributing to tumor aggressiveness through sustained proliferation, invasion into normal brain tissue, and suppression of anticancer immunity [64]. Malta et al. developed several novel stemness indices to assess the degree of oncogenic dedifferentiation based on transcriptomic and epigenetic features [65]. In this study, we primarily explored the relationship between PANoptosis enrichment and the mRNA expression-based stemness index (mRNAsi). We found that a higher mRNAsi, which is negatively correlated with tumor pathology and clinical features in gliomas [65], was associated with a lower PANoScore (Fig. 5A-B). Recently, Wang et al. defined three tumor-intrinsic transcriptional subtypes of GBM-IDHwt: proneural (PN), mesenchymal (MES), and classical (CL), which are partly shaped by the tumor immune microenvironment [66, 67]. Previous studies have reported that the MES subtype is more aggressive than the PN and CL subtypes [67, 68]. In our work, we observed that GBMs with a high PANoScore are more likely to be classified as the MES subtype (Fig. 5C-D). Currently, scRNA-seq analysis has classified GBM cells into four states: astrocyte-like (AC-like), mesenchymal-like (MES-like), neural-progenitor-like (NPC-like), and oligodendrocyte-progenitor-like (OPC-like), to further understand the intratumoral heterogeneity of GBM. This analysis revealed that MES GBMs generally contain more AC-like and MES-like cells, which include a significant number of proliferating cells, reflecting their aggressive nature [28]. In our study, we found that GBM cells with a high PANoScore tended to be grouped as AC-like and MES-like cells (Fig. 5E), and they exhibited a higher PANoScore compared with NPC-like and OPC-like cells (Fig. 5F). Moreover, GBMs with a high PANoScore contained substantial subsets of AC-like and MES-like cells (Fig. 5G). Additionally, genetic alterations in certain oncogenes may promote specific GBM cellular states by enhancing growth and/or inducing state transitions. Oncoplot genomic analysis showed that EGFR was the most frequently altered gene (71.9%) among those with significant mutational differences between the high and low PANoScore groups (Fig. 5H). EGFR overexpression can drive astrocyte proliferation and induce an AC-like program in GBM [28], further implying that PANoptosis enrichment is related not only to tumor progression but also to the distribution of cellular states within GBM. Interestingly, analyses of the GDSC datasets indicate that the group with a higher PANoScore showed increased sensitivity to several EGFR-targeted drugs, including erlotinib and gefitinib (Fig. 5I-J). Tumor cells, along with the tumor microenvironment, create a highly complex milieu that ultimately promotes the transcriptomic adaptability and aggressiveness of GBM cells [67, 69]. GSEA revealed that several immune-related pathways, such as the IFNα/γ response, JAK/STAT signaling, and TNFα signaling, are enriched in high PANoScore GBM (Fig. 5K-L and Table S5). This finding implies that immune signals may be involved in regulating the enrichment of PANoptosis and that PANoptosis may also lead to remodeling of the tumor microenvironment.

### Interconnection between PANoptosis enrichment and tumor immune dynamics

Next, we explored the relationship between PANoptosis enrichment and tumor immunity. First, we identified coexpression modules through WGCNA in the TCGA-GBM-IDHwt (Fig. 6A-B) and CPTAC-GBM-IDHwt (Supplement Fig. 9A-B) cohorts. Based on the correlation analysis between modules and the PANoScore (Fig. 6C, Supplement Fig. 9C, and Table S6), the modules with |r| > 0.6 and *P* < 0.05 were considered to be highly associated with PANoptosis enrichment. After that, we selected the modules with the greatest positive correlation with the PANoScore: the turquoise module in TCGA-GBM-IDHwt (*r* = 0.711, *P* = 2.34E-24) and the turquoise module in CPTAC-GBM-IDHwt (*r* = 0.766, *P* = 3.80E-19). The 267 common coexpressed genes from the two turquoise modules (Supplement Fig. 9D and Table S7) were subsequently subjected to GO/KEGG enrichment analyses. Consistent with the GSEA results (Fig. 5K-L and Table S5), in addition to processes associated with cell death, the PANoScore was also significantly correlated with a series of immune system processes and pathways (Fig. 6D and Table S8). These findings imply that PANoptosis may play an important role in regulating cancer immunological processes.

**Fig. 9:**
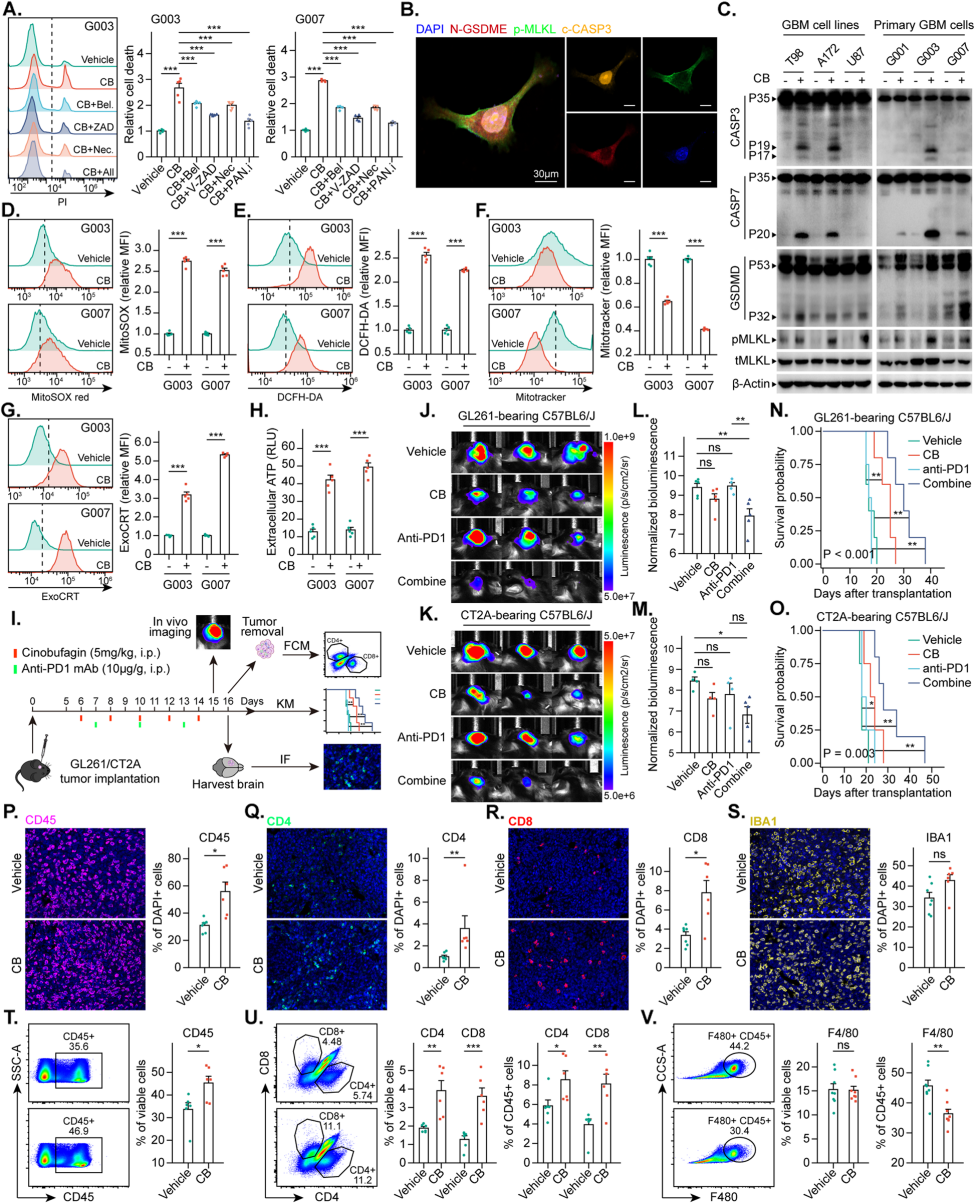
Cinobufagin Induces Immunogenic PANoptosis and Potentiates GBM to Anti-PD-1 therapy.** A **Propidium iodide (PI) staining showing the death of G003 and G007 cells treated with cinobufagin (CB), pyroptosis inhibitor (Belnacasan), apoptosis inhibitor (V-ZAD-FMK), and necroptosis inhibitor (Necrostatin) for 24 h. **B **Multiplex immunohistochemistry (mIHC) of N-GSDME, c-CASP3 and p-MLKL in G003 cells treated with CB for 24 h. **C **Western blot (WB) analysis of PANoptosis markers (CASP3/7, c-CASP3/7, GSDMD, GSDME, MLKL, and p-MLKL) in GBM cell lines and primary GBM cells. **D-F **Flow cytometry showing mitochondrial stress markers in G003 and G007 cells following CB treatment: (**D**) MitoSox-red for mitochondrial superoxide, (**E**) DCFH-DA for intracellular ROS, and (**F**) MitoTracker for mitochondrial mass. **G** Flow cytometry analysis of the externalization of calreticulin to the cell membrane (ExoCRT). **H** Release of extracellular ATP quantified via an ADP/ATP ratio assay kit. **I **Schematic of the in vivo experimental design using GL261 and CT2A cells in C57BL/6J mice. **J-K **In vivo imaging on day 15 postimplantation for GL261 and CT2A tumors. **L-M **Quantitative bioluminescence analysis for (**J**) and (**K**), respectively. *N* = 4–5 mice/group. **N-O** Kaplan-Meier survival curves for GL261 and CT2A tumor-bearing mice under different treatments. **P-S **Immunofluorescence (IF) showing infiltration of CD45^+^, CD4^+^, CD8^+^, and IBA1^+^ cells in CT2A tumors. *N* = 6–7 mice/group. **T **Flow cytometry of CD45^+^ cells in GL261 tumors. *N* = 6 mice/group. **U-V **Flow cytometry of CD4^+^/CD8^+^ T cells and F4/80^+^ cells in GL261 tumors, showing absolute infiltration and relative proportion among CD45^+^ cells

To further explore the correlation between PANoptosis and tumor immunology, TIP analysis was performed to profile the status of antitumor immunity in GBM-IDHwt. We calculated 23 anticancer immune activity scores across the seven steps of the cancer-immunity cycle and the overall anticancer immune activity scores for all GBM samples (Table S9). We found that GBM patients with a high PANoScore exhibited increased activity in the release of cancer cell antigens (step 1), recruitment of immune cells (step 4), and infiltration of immune cells into tumors (step 5); however, there was dampened activity in the recognition and killing of cancer cells (steps 6 and 7). Overall, the high PANoScore group demonstrated enhanced activity of anti-cancer immunity compared to the low PANoScore group (Fig. 6E and Supplement Fig. 10A). Furthermore, correlation analysis indicated that PANoptosis enrichment was positively correlated with the recruitment of various immune cells, including T cells, NK cells, dendritic cells, and monocytes/macrophages (Fig. 6F). These results suggest that although PANoptosis enrichment does not contribute to the final killing of tumor cells, it significantly promotes the initiation and processing phases of the antitumor immune response. IPS analysis was performed to reflect the immunophenotypes and tumor escape mechanisms (Table S10) and demonstrated that the PANoScore was positively associated with scores for antigen processing and effector cells but negatively associated with scores for suppressor cells and checkpoints (Fig. 6G). This further implies that PANoptosis enrichment may enhance antitumor immunity in GBM-IDHwt patients. Through ESTIMATE analysis, we discovered that the PANoScore was significantly positively correlated with the immune score in GBM-IDHwt patients. Additionally, when TIMER, MCPCounter, EPIC, and xCell methods were used, a clear positive correlation was verified between the PANoScore and the infiltration of CD8^+^ T cells (Supplement Fig. 10B-C). Although PANoptosis enrichment promoted the infiltration of immune cells, particularly CD8^+^ T cells, we found that the expression of some immune checkpoints (such as PD-L1, PD-1, and CTLA4), which function as “brakes” on T cell activation, also increased with higher PANoScore (Supplement Fig.10D). These findings suggest that GBMs with a high PANoScore may have a better opportunity for immunotherapies and are more likely to respond to immune checkpoint inhibition.

**Fig. 10:**
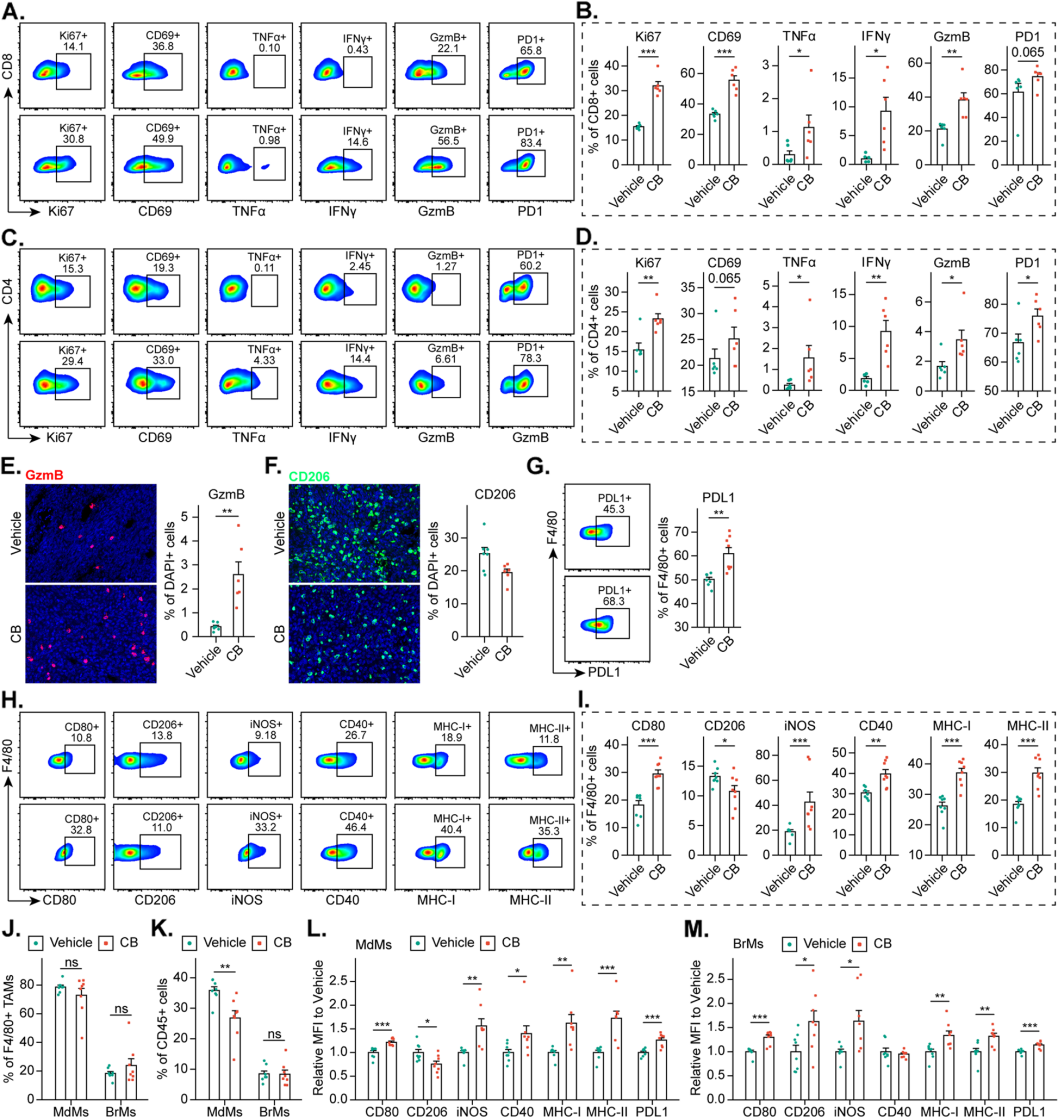
Cinobufagin promotes T Cell activation and TAMs Reprogramming.** A-D **Percentages of Ki67^+^, CD69^+,^ TNFα^+^, IFNγ^+^, GzmB^+^, and PD1^+^ of total CD8^+^ and CD4^+^ T cells in GL261 tumors identified by flow cytometry. *N* = 6 mice/group. **E-F **IF staining of GzmB^+^ and CD206^+^ cells in CT2A tumors. *N* = 6–7 mice/group. **G **Percentages of PD-L1^+^ cells among total F4/80^+^ cells in GL261 tumors identified by flow cytometry. **H-I **The percentages of CD80^+^, CD206^+^, iNOS^+^, CD40^+^, MHC-I^+^, and MHC-II^+^ cells among total F4/80^+^ cells in GL261 tumors identified by flow cytometry. **J-K **Percentages of monocyte-derived macrophages (MdMs) and brain-resident microglia (BrMs) among F4/80^+^ cells and CD45^+^ cells in GL261 tumors identified by flow cytometry, respectively. **L-M **Mean fluorescence intensity (MFI) of CD80, CD206, iNOS, CD40, MHC-I, and MHC-II and PD-L1 in MdMs and BrMs identified in GL261 tumors via flow cytometry

### Evaluating enhanced PANoptosis in GBM immunotherapy

Immunotherapies, such as PD-1/PD-L1 blockade, have revolutionized cancer treatment; however, their efficacy in GBM remains limited [70, 71]. Therefore, we investigated the relationship between PANoptosis enrichment and response to immunotherapy. Firstly, SubMap and TIDE machine learning methods were employed to predict the impact of the PANoScore on immunotherapy response and efficacy (Fig. 7A). SubMap analysis indicated that GBM patients with a high PANoScore may exhibit greater sensitivity to anti-PD-1 therapy (Fig. 7A). TIDE analysis revealed that responders to immunotherapy had higher PANoScore than nonresponders, with TIDE scores decreasing as the PANoScore increased (Fig. 7B-E). We subsequently utilized four clinical immunotherapy cohorts (PRJNA482620, PRJEB23709, GSE91061, and GSE35640) for validation, revealing a significant trend toward higher PANoScore in responders than in nonresponders (Fig. 7F-I). Furthermore, patients with high PANoScores presented a clear trend toward longer progression-free survival (PFS) and overall survival (OS) compared to those with low PANoScores (Fig. 7J-O). These findings suggest that PANoptosis enrichment may be correlated with improved responses and enhanced efficacy of immunotherapies. TIDE analysis of the TCGA-GBM-IDHwt and CPTAC-GBM-IDHwt cohorts (Fig. 7P-Q) showed a significant decrease in the scores of myeloid-derived suppressor cells (MDSCs) and M2-type tumor-associated macrophages (M2-TAMs) in the high PANoScore group, indicating that PANoptosis enrichment may help reverse the highly immunosuppressive tumor microenvironment. Additionally, the CD8 and IFNG scores increased, whereas the T cell exclusion scores decreased, suggesting that PANoptosis enrichment promotes the infiltration of cytotoxic T cells, which are crucial for antitumor immune responses. However, the scores for immune checkpoints (CD274, which encodes PD-L1) and T cell dysfunction were also significantly elevated. These findings may explain why PANoptosis enrichment is associated with an improved prognosis following anti-PD-1/PD-L1 therapy in GBM. To further investigate this conjecture, TIP, xCell, and quanTIseq analyses were conducted in the GBM immunotherapy cohort (PRJNA482620-GBM-IDHwt). We found that the PANoScore was significantly positively correlated with overall anticancer immune activity, the release of cancer cell antigens, the recruitment of CD8^+^ T cells, the recruitment of monocytes/macrophages, and the infiltration of M1-type tumor-associated macrophages (M1-TAMs) (Supplement Fig. 11). Additionally, TIDE analysis also suggested that the PANoScore was significantly negatively correlated with the M2-TAM infiltration and positively correlated with the IFNG score (Fig. 7R). In conclusion, enhanced PANoptosis enrichment may sensitize GBM to anti-PD-1/PD-L1 therapies by promoting CD8^+^ T cell recruitment and infiltration into the tumor, as well as facilitating M1-like macrophage activation while suppressing polarization towards M2-like.

### Identifying potential small-molecule activators of PANoptosis in GBM

Given the scarcity of definitive PANoptosis activators, we predicted potential small-molecule compounds based on PANoScore. First, we conducted differential expression gene (DEG) analyses between the high and low PANoScore groups in the TCGA-GBM-IDHwt, CPTAC-GBM-IDHwt, and PRJNA482620-GBM-IDHwt cohorts (Fig. 8A-C). A total of 59 upregulated and 44 downregulated common genes were identified through the intersection of DEGs across these cohorts (Fig. 8D). Functional enrichment analysis revealed that these common DEGs are enriched primarily in pathways such as TNF-α signaling via NFKB, IL6-JAK-STAT3 signaling, inflammatory response, apoptosis, interferon gamma response, and hypoxia (Fig. 8E and Table S11). Finally, candidate small-molecule compounds and their mechanisms of action were predicted through the CMap database and MOA analysis based on these 103 common DEGs. The top 30 compounds with scores exceeding 90 are shown in Fig. 8F and Table S12. Notably, several ATPase inhibitors (including ouabain, helveticoside, cinobufagin, strophanthidin, proscillaridin, digoxin, and digitoxin) and PKC activators (including prostratin, ingenol, and phorbol-12-myristate-13-acetate) have demonstrated significant potential for inducing PANoptosis. These findings offer new insights and references for developing novel drugs aimed at enhancing PANoptosis in GBM.

### Cinobufagin induces immunogenic PANoptosis and potentiates GBM to anti-PD-1 therapy

Cinobufagin (Supplement Fig. 12A), an active compound derived from Venenum bufonis, is a traditional Chinese medicine recognized for inducing apoptosis [20, 21]. Our previous research demonstrated that cinobufotalin, an analog of cinobufagin, effectively blocks the cell cycle and induces cell death in glioma cells [72]. However, its ability to activate immunogenic PANoptosis in GBM cells and modulate antitumor immunity remains poorly understood. CCK8 and PI staining assays revealed that cinobufagin significantly reduced GBM cell viability, leading to cell death (Supplement Fig. 12B-C and Fig. 9A). Multicolor immunofluorescence demonstrated that cinobufagin enhanced the expression of cleaved N-terminal gasdermin-E (N-GSDME, a pyroptosis executor), cleaved caspase-3 (c-CASP3, an apoptosis executor), and phosphorylated mixed lineage kinase domain-like protein (p-MLKL, a necroptosis executor) in GBM tumor cells. Nuclear translocation of c-CASP3 and membrane aggregation of p-MLKL were also observed (Fig. 9B). Western blot analysis further confirmed that N-GSDMD/E, c-CASP3/7, and p-MLKL were significantly upregulated in various GBM tumor cells following stimulation with cinobufagin (Fig. 9C). These results confirm that cinobufagin can activate PANoptosis in GBM tumor cells. We then used MitoSox-red, DCFH-DA, and MitoTracker probes to assess mitochondrial superoxide levels, intracellular ROS, and mitochondrial mass. Our findings indicated that treatment with cinobufagin led to a significant increase in both mitochondrial superoxide levels and intracellular ROS, accompanied by a marked reduction in the mitochondrial mass in GBM tumor cells (Fig. 9D-F). These findings suggest that mitochondrial damage and oxidative stress may contribute to cinobufagin-induced PANoptosis in GBM cells. To further investigate whether the cell death induced by cinobufagin has an immunostimulatory nature, we assessed the expression of ICD drivers, including the externalization of calreticulin to the cell membrane (ExoCRT) and the release of intracellular ATP. Cinobufagin treatment increased ExoCRT and extracellular ATP levels in G003 and G007 cells (Fig. 9G-H), suggesting enhanced tumor immunogenicity alongside cell death. We next asked: could cinobufagin modulate cancer cells in vivo within the context of a complex TME? Specifically, whether cinobufagin could synergistically enhance the efficacy of GBM immunotherapy. To investigate this, we utilized two mouse glioblastoma models: transplanted GL261 and CT2A tumors (Fig. 9I). The in vivo imaging and survival analysis results demonstrated that cinobufagin alone can inhibit tumor growth and prolong the survival of GL261- and CT2A-bearing mice. The combination of cinobufagin with anti-PD1 monoclonal antibodies resulted in a significantly more pronounced therapeutic effect (Fig. 9J-O). Importantly, these treatments did not affect the body weight of C57BL/6J mice (Supplement Fig. 12D-E), nor did they lead to elevated serum levels of creatine kinase, albumin, alkaline phosphatase (ALP), or aspartate aminotransferase (AST) (markers of heart, kidney and liver damage) (Supplement Fig. 12F). Moreover, histological evaluation of the heart, liver, spleen, lungs, and kidneys of the treated mice revealed no signs of toxicity (Supplement Fig. 12G). Next, we evaluated cinobufagin’s effect on immune cell populations in the TME. Immunofluorescence (IF) staining of CT2A tumor tissues revealed that, compared with vehicle treatment, cinobufagin treatment significantly increased the infiltration of CD45^+^ immune cells, with a particularly notable increase in CD4^+^ T cells and CD8^+^ T cells. However, no significant difference was observed in the overall proportion of IBA1^+^ tumor-associated microglia/macrophages (TAMs) (Fig. 9P-S). By flow cytometry (Supplement Fig. 12H), we found that in GL261 tumors, the proportions of CD45^+^ immune cells, CD4^+^ T cells and CD8^+^ T cells increased in the cinobufagin treatment group, which was consistent with the results of IF staining of CT2A tumors. Among the CD45^+^ immune cells, the proportions of CD4^+^ and CD8^+^ T cells remained elevated, whereas F4/80^+^ TAMs exhibited a notable decline (Fig. 9T-V). In conclusion, cinobufagin has the potential to enhance the effectiveness of GBM immunotherapy and inhibit tumor progression by inducing immunogenic PANoptosis and promoting antitumor immune responses. This highlights its role as a modulator of the tumor microenvironment and a promising therapeutic option for glioma.

### Cinobufagin promotes T cell activation and TAMs reprogramming

T cells play a central role in antitumor immunity. We then characterized the CD8^+^ and CD4^+^ T cells populating GL261 tumors and spleens. Flow cytometry analysis revealed an increase in the proliferative (Ki67^+^), activated (CD69^+^), and cytotoxic (TNFα^+^, IFNγ^+^, GzmB^+^) CD8^+^ and CD4^+^ T cells in tumor tissues (Fig. 10A-D) and spleens (Supplement Fig. 12I-L) of GL261 tumor-bearing mice treated with cinobufagin, compared to the vehicle-treated group. Similarly, there was a significant increase in the accumulation of GzmB^+^ cells in CT2A tumor tissues following cinobufagin treatment (Fig. 10E). These findings underscore the role of the adaptive immune system in driving the response to cinobufagin treatment. In GBM, TAMs, from brain-resident microglia (BrMs) and monocyte-derived macrophages (MdMs), are protumorigenic and associated with immunosuppression. Therefore, we investigated whether TAM polarization was altered in cinobufagin-treated tumors by initially assessing the expression of markers associated with the M1-like (CD80) tumor-suppressive phenotype and the M2-like (CD206) tumor-promoting phenotype. IF staining of CT2A tumors revealed that cinobufagin treatment upregulated the M1-like program while downregulating the M2-like program (Fig. 10F), which was corroborated by flow cytometry analysis of GL261 tumors (Fig. 10H-I). We further analyzed the tumoricidal activity of TAMs by comparing iNOS and CD40 expression and evaluated their activation and antigen-presenting potential by analyzing MHC-I and MHC-II levels. Notably, we observed a significant increase in the proportions of iNOS^+^, CD40^+^, MHC-I^+^, and MHC-II^+^ cells among F4/80^+^ TAMs (Fig. 10H-I). Since tumor-associated MdMs and BrMs each contribute to the immunosuppressive microenvironment in GBM but differ in quantity, spatial distribution, and transcriptional programs, we further characterized the features of each cell type. We found that approximately 80% of TAMs were MdMs (CD45^high^ CD11b^+^ F4/80^+^), whereas only approximately 20% were BrMs (CD45^low^ CD11b^+^ F4/80^+^). Although cinobufagin slightly increased the proportion of BrMs relative to that of MdMs, this change did not reach statistical significance (Fig. 10J). From the perspective of CD45^+^ immune cells, there was a significant decrease in the proportion of MdMs (Fig. 10K), suggesting that the change in MdMs quantity may primarily contribute to the overall reduction of TAMs induced by cinobufagin. Although the changes in the expression of CD206 and CD40 in MdMs and BrMs were inconsistent, the upregulation of CD80, iNOS, MHC-I, and MHC-II in both cell types suggested that cinobufagin exerts a universally broad effect on the polarization states, tumoricidal activity, and antigen presentation capacity of both MdMs and BrMs (Fig. 10L-M). Finally, we observed a significant upregulation of PD-1 expression in T cells (Fig. 10A-D) and PD-L1 expression in TAMs (Fig. 10G, L-M). This finding indicates that while cinobufagin activates antitumor immunity, it also triggers the activation of the PD-1/PD-L1 immune checkpoint axis, thereby initiating an immune “brake”.

## Discussion

PANoptosis, a novel form of programmed cell death integrating pyroptosis, apoptosis, and necroptosis, has emerged as a pivotal mechanism in cancer biology [6, 7]. This synergistic process not only facilitates cell death but also plays a crucial role in modulating immune responses within the TME [14–16]. While individual pathways of cell death have been extensively studied [73–77], PANoptosis as an integrated mechanism remains underexplored in gliomas. Prior studies suggest that PANoptosis-related dysregulation contributes to glioma aggressiveness and therapy resistance [17–19], but gaps remain in understanding its clinical implications.

In this study, PANoRGs were selected based on their pivotal roles in PANoptosis. Key executors such as CASP3, CASP7, GSDMD, and MLKL mediate apoptosis, pyroptosis, and necroptosis, respectively. Essential components forming the PANoptosome scaffold, including CASP1, CASP6, CASP8, FADD, PYCARD, RIPK3, and NLRP3, were included for their critical roles in assembly and function. Upstream sensors like ZBP1, AIM2, and RIPK1, along with regulators identified in cancer-related PANoptosis studies, such as IRF1, ADAR, MAP3K7, and NFS1, were also incorporated for their significant regulatory roles. Together, these genes form the foundation of the gene set used in this study, underscoring their critical roles in PANoptosis and GBM. We identified distinct PANoptosis-related molecular subtypes (PANoSubtypes) in gliomas, characterized by varying PANoptosis enrichment scores (PANoScores) based on PANoRGs. These PANoSubtypes were significantly associated with clinical features and patient survival. Gliomas with higher PANoScores exhibited more aggressive phenotypes, poorer survival outcomes, and unique molecular characteristics. Importantly, we developed a prognostic model combining PANoScore and clinical variables, demonstrating high predictive accuracy across independent glioma cohorts. This robust scoring system underscores PANoScore as a novel biomarker, providing insights into glioma progression and patient outcomes. These findings emphasize the clinical relevance of PANoptosis patterns in gliomas, establishing a foundation for precision oncology.

The immune microenvironment of GBM is profoundly immunosuppressive, characterized by limited cytotoxic T cell infiltration and the dominance of M2-like TAMs, collectively creating an immune “cold” phenotype. These features contribute to the limited efficacy of immunotherapies, such as immune checkpoint inhibitors, in GBM [3–5]. PANoptosis, as a highly immunogenic form of cell death, offers a unique avenue to modulate the TME and enhance immune responses [15, 16]. Our results demonstrate that PANoScore is closely linked to immune activity within the GBM microenvironment. High PANoScore tumors were associated with increased antigen release, enhanced antigen presentation, and greater recruitment of immune cells, including CD8^+^ T cells and dendritic cells, although these tumors also displayed diminished activity in tumor cell recognition and killing. Additionally, PANoScore was positively correlated with immune checkpoint expression, such as PD-L1 and CTLA-4, suggesting a compensatory upregulation of immune evasion mechanisms in response to heightened immune activity. Clinically, high PANoScore tumors showed better responses to immune checkpoint inhibitors, with improved progression-free and overall survival, underscoring PANoScore’s predictive value for immunotherapy efficacy. These findings highlight the dual nature of PANoScore in GBM’s immune dynamics: enhancing immune activation while concurrently revealing immune evasion vulnerabilities, primarily through immune checkpoint upregulation. Targeting PANoptosis and leveraging its immunogenic potential in conjunction with checkpoint inhibitors to counteract immune suppression could provide a novel approach to convert immune “cold” GBM tumors into “hot” ones, improving immunotherapeutic outcomes.

Cinobufagin (CB), a bioactive compound derived from traditional Chinese medicine, has garnered attention for its antitumor properties, particularly its ability to induce various forms of programmed cell death [20–22]. While previous studies demonstrated CB’s ability to trigger apoptosis, pyroptosis, or necroptosis, its role in PANoptosis activation and its influence on the TME remain underexplored. In this study, we confirmed that cinobufagin robustly induces PANoptosis in GBM cells, triggering pyroptosis, apoptosis, and necroptosis via the upregulation of key executors such as cleaved caspase-3/7, N-GSDMD, and p-MLKL. This activation was accompanied by mitochondrial damage and heightened reactive oxygen species (ROS) production, which contribute to the compound’s cytotoxic effects. Moreover, CB increased the immunogenicity of GBM cells, as evidenced by elevated ATP release and calreticulin externalization, hallmark features of ICD. Our in vivo experiments further revealed that CB synergizes with anti-PD-1 therapy to suppress tumor growth, improve survival, and modulate the immune microenvironment. Specifically, CB promoted the infiltration and activation of CD4^+^ and CD8^+^ T cells while reprogramming TAMs to a tumoricidal M1-like phenotype. These effects were achieved alongside increased expression of immune-stimulatory molecules (e.g., MHC-II, CD40, CD80) and reduced levels of immunosuppressive markers like CD206. However, the observed upregulation of immune checkpoints, such as PD-1 and PD-L1, highlights the necessity of combining CB with checkpoint inhibitors to overcome adaptive resistance mechanisms. Notably, these therapeutic effects were achieved without notable toxicity, underscoring CB’s safety and efficacy as an adjunct to immunotherapy. In summary, CB demonstrates significant potential to enhance GBM immunotherapy through the induction of immunogenic PANoptosis and modulation of the immune microenvironment. By converting GBM’s immune “cold” phenotype into a “hot” one, CB effectively sensitizes tumors to immune checkpoint inhibitors.

Despite the promising findings, this study has several limitations. First, the PANoScores of GL261 and CT2A models following CB treatment were not directly quantified and compared, which could provide additional insights into PANoptosis variations across models. Second, the exact upstream mechanisms by which CB induces PANoptosis in GBM cells remain uncertain. While our prior research shows Cinobufotalin binds to inosine monophosphate dehydrogenase 1 (IMPDH1) to induce apoptosis [71], whether this mechanism is also involved in PANoptosis requires further validation. Lastly, while preclinical models demonstrated CB’s synergy with anti-PD-1 therapy, clinical translation remains challenging due to the immunosuppressive GBM microenvironment and variability in patient responses. Further studies are needed to optimize dosing, assess safety, and validate efficacy in clinical trials.

## Conclusions

This study underscores the critical role of PANoptosis in transforming “cold” gliomas into “hot” tumors, thereby enhancing their responsiveness to immunotherapy. Gliomas with higher PANoScore exhibit significant immune cell infiltration, particularly effector CD8^+^ T cells and TAMs reprogrammed to a tumoricidal M1-like phenotype. PANoptosis activation, driven notably by the small molecule cinobufagin, induces immunogenic cell death, boosts antitumor immune responses, and sensitizes gliomas to anti-PD-1 therapy. Importantly, while gliomas with a high PANoScore show elevated immune checkpoint expression, potentially limiting T cell activity, targeting PANoptosis may mitigate this immune suppression. These findings suggest that combining PANoptosis activators with immune checkpoint inhibitors could provide a synergistic strategy to overcome immune evasion, reprogram the tumor microenvironment, and improve therapeutic outcomes for glioma patients.

## Supplementary Information

Supplementary Material 1.

Supplementary Material 2.

Supplementary Material 3.

Supplementary Material 4.

Supplementary Material 5.

Supplementary Material 6.

Supplementary Material 7.

Supplementary Material 8.

Supplementary Material 9.

Supplementary Material 10.

Supplementary Material 11.

Supplementary Material 12.

Supplementary Material 13.

## Data Availability

No datasets were generated or analysed during the current study.

## Abbreviations

AC-like: Astrocyte-like
ALP: Alkaline phosphatase
AST: Aspartate aminotransferase
ATP: Adenosine triphosphate
BrM: Brain-resident microglia
BSA: Bovine serum albumin
CB: Cinobufagin
CCK-8: Cell Counting Kit-8
CDF: Cumulative distribution function
CGGA: Chinese glioma genome atlas
CL: Classical
CMap: Connectivity map
CPTAC: Clinical proteomic tumor analysis consortium
DCA: Decision curve analysis
DEG: Differentially expressed gene
ExoCRT: Externalization of calreticulin to the cell membrane
GBM: Glioblastoma multiforme
GBM-IDHwt: IDH-wildtype glioblastoma multiforme
GDSC: Genomics of drug sensitivity in cancer
GO: Gene ontology
GSCs: Glioblastoma stem cells
GSEA: Gene set enrichment analysis
HE: Hematoxylin and eosin
ICD: Immunogenic cell death
ICIs: Immune checkpoint inhibitors
IF: Immunofluorescence
IPS: Immunophenoscore
KEGG: Kyoto encyclopedia of genes and genomes
LGG: Low-grade glioma
M1-TAMs: M1-like tumor-associated macrophages
M2-TAMs: M2-type tumor-associated macrophages
MdM: Monocyte-derived macrophage
MDSC: Myeloid-derived suppressor cells
MES: Mesenchymal
MES-like: Mesenchymal-like
mIHC: Multiplex immunohistochemistry
MOA: Mode-of-action
mRNAsi: mRNA-based stemness index
NPC-like: Neural-progenitor-like
OPC-like: Oligodendrocyte-progenitor-like
OS: Overall survival
PANoRGS: PANoptosis-related genes
PANo-RRS: PANoptosis-related risk score
PANoScore: PANoptosis enrichment score
PANoSubtypes: PANoptosis-related molecular subtypes
PCA: Principal component analysis
PCD: Programmed cell death
PI: Propidium iodide
PN: Proneural
PPI: Protein-protein interaction
RNA-seq: RNA sequencing
ROC: Receiver operating characteristic
ROS: Reactive oxygen species
scRNA-seq: Single-cell RNA sequencing
ssGSEA: Single-sample gene set enrichment analysis
TAMs: Tumor-associated macrophages
TCGA: The cancer genome atlas
TIDE: Tumor immune dysfunction and exclusion
TIP: Tracking tumor immunophenotype
TME: Tumor microenvironment
WGCNA: Weighted correlation network analysis
WB: Western blot
