# Supplementary figures and images for: Integrative analysis of immunogenic PANoptosis and experimental validation of cinobufagin-induced activation to enhance glioma immunotherapy
Source: J Exp Clin Cancer Res. 2025 Feb 3;44:35. doi: 10.1186/s13046-025-03301-1 (PMC11789371; doi:10.1186/s13046-025-03301-1)

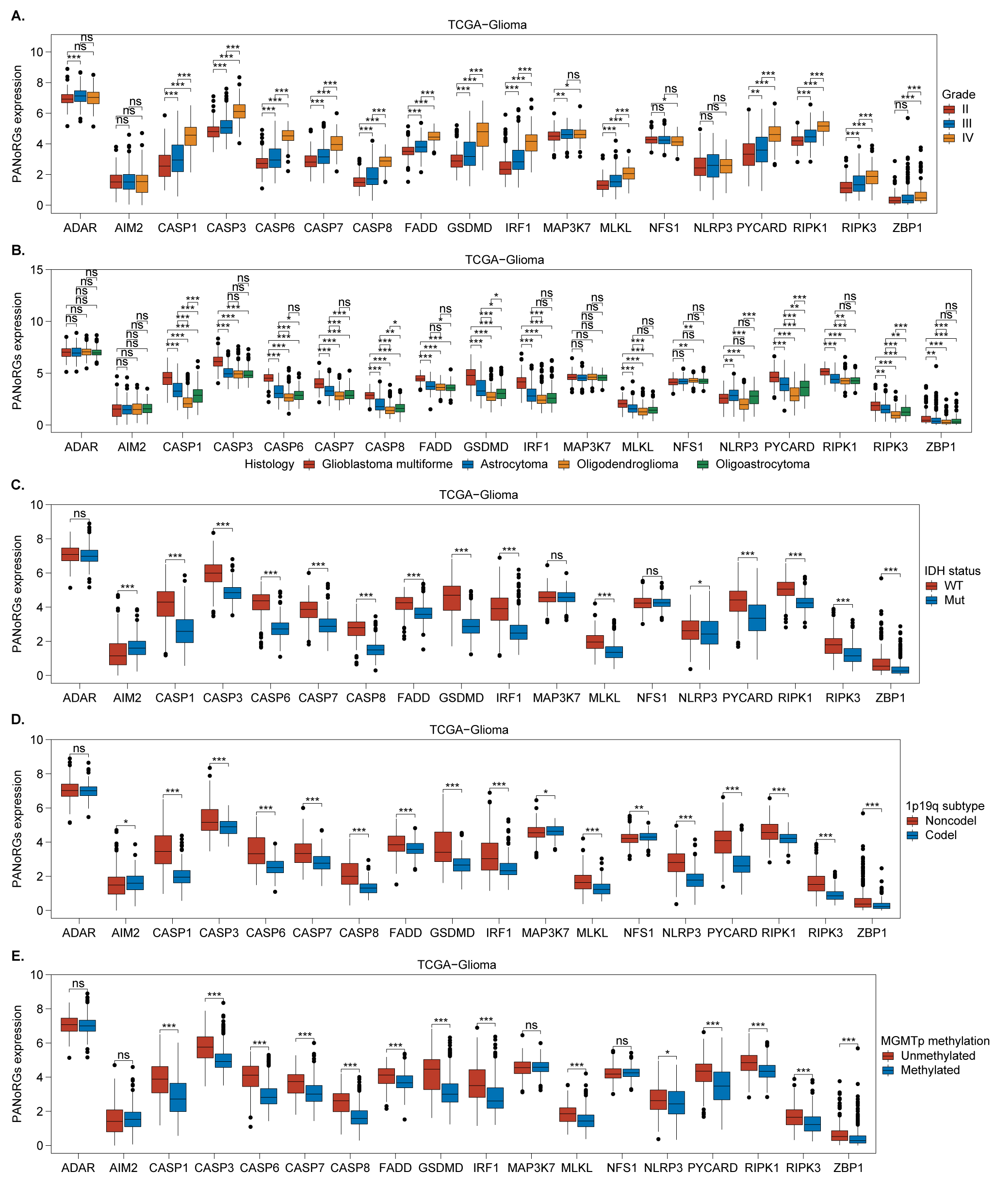

Supplement: Supplementary file 2 — Supplementary Material 2. [file 13046_2025_3301_MOESM2_ESM.tif]

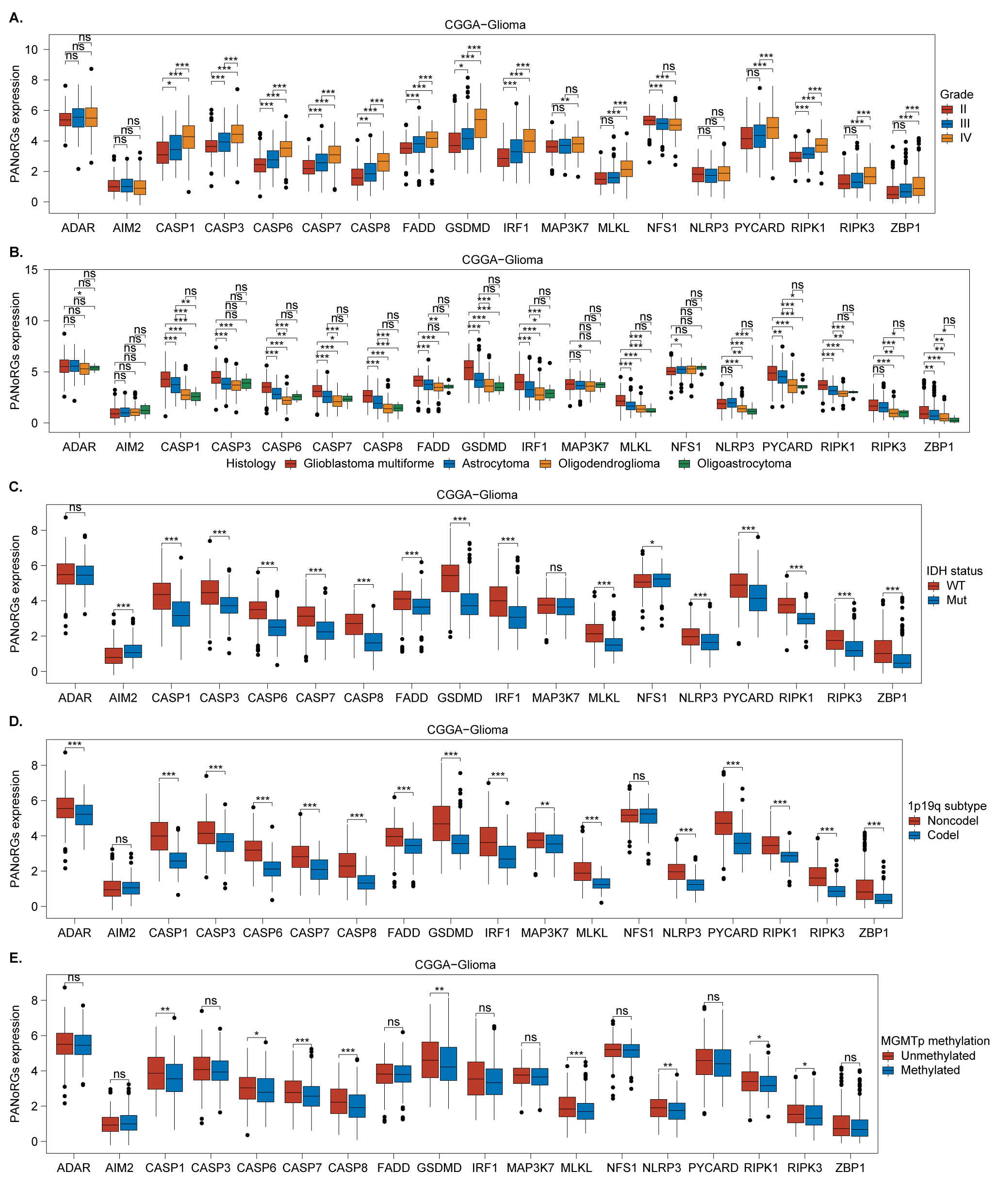

Supplement: Supplementary file 3 — Supplementary Material 3. [file 13046_2025_3301_MOESM3_ESM.tif]

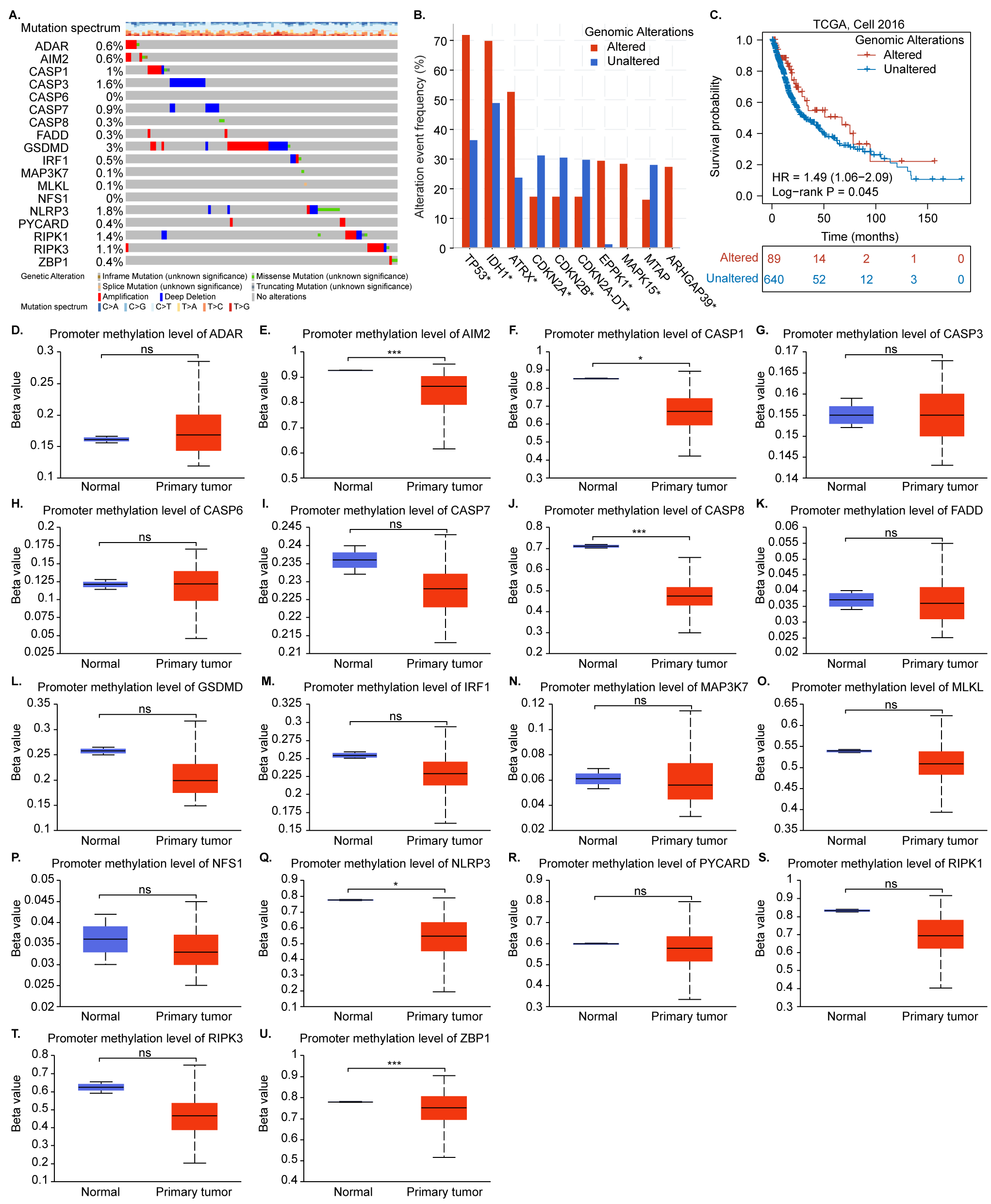

Supplement: Supplementary file 4 — Supplementary Material 4. [file 13046_2025_3301_MOESM4_ESM.tif]

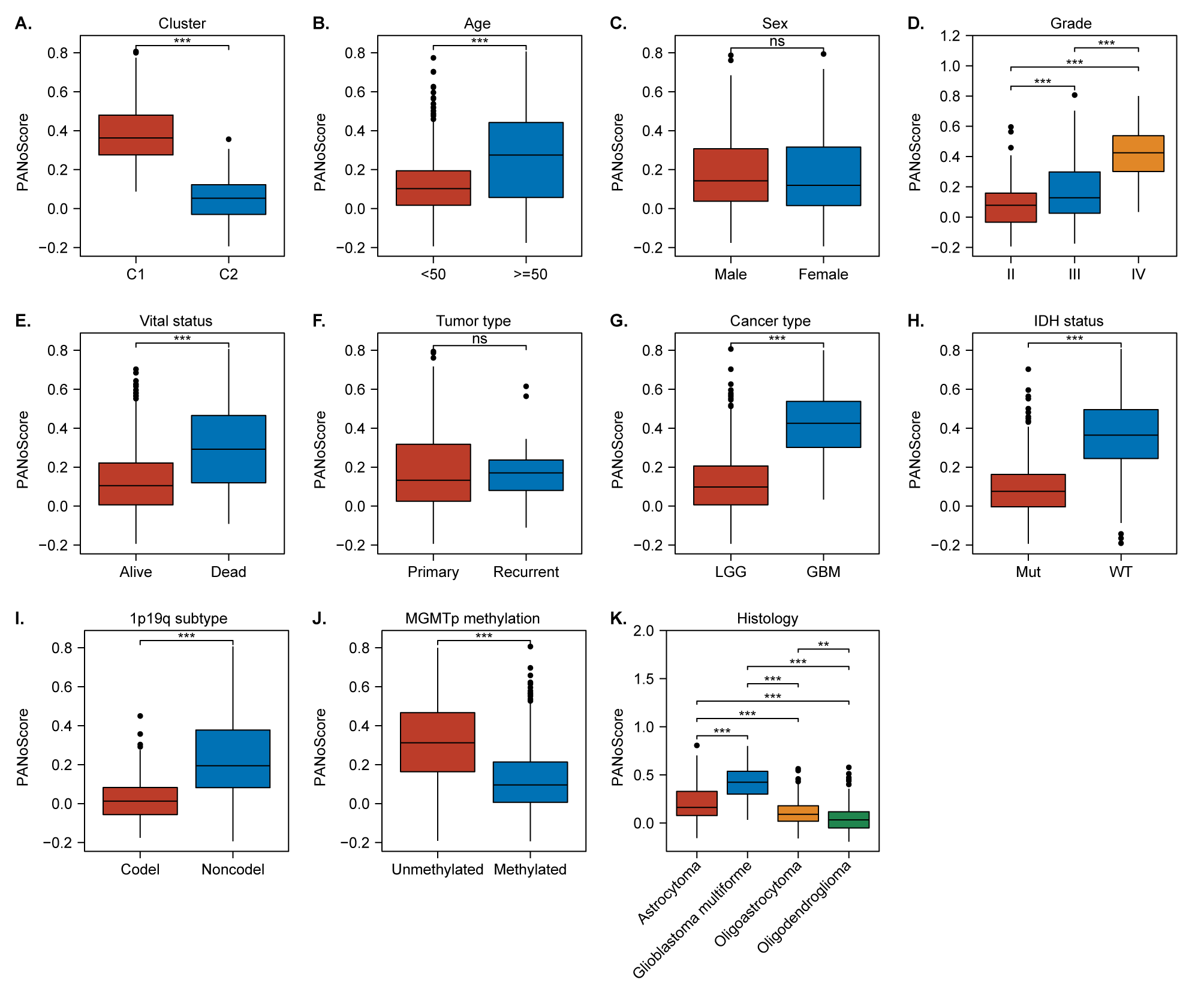

Supplement: Supplementary file 5 — Supplementary Material 5. [file 13046_2025_3301_MOESM5_ESM.tif]

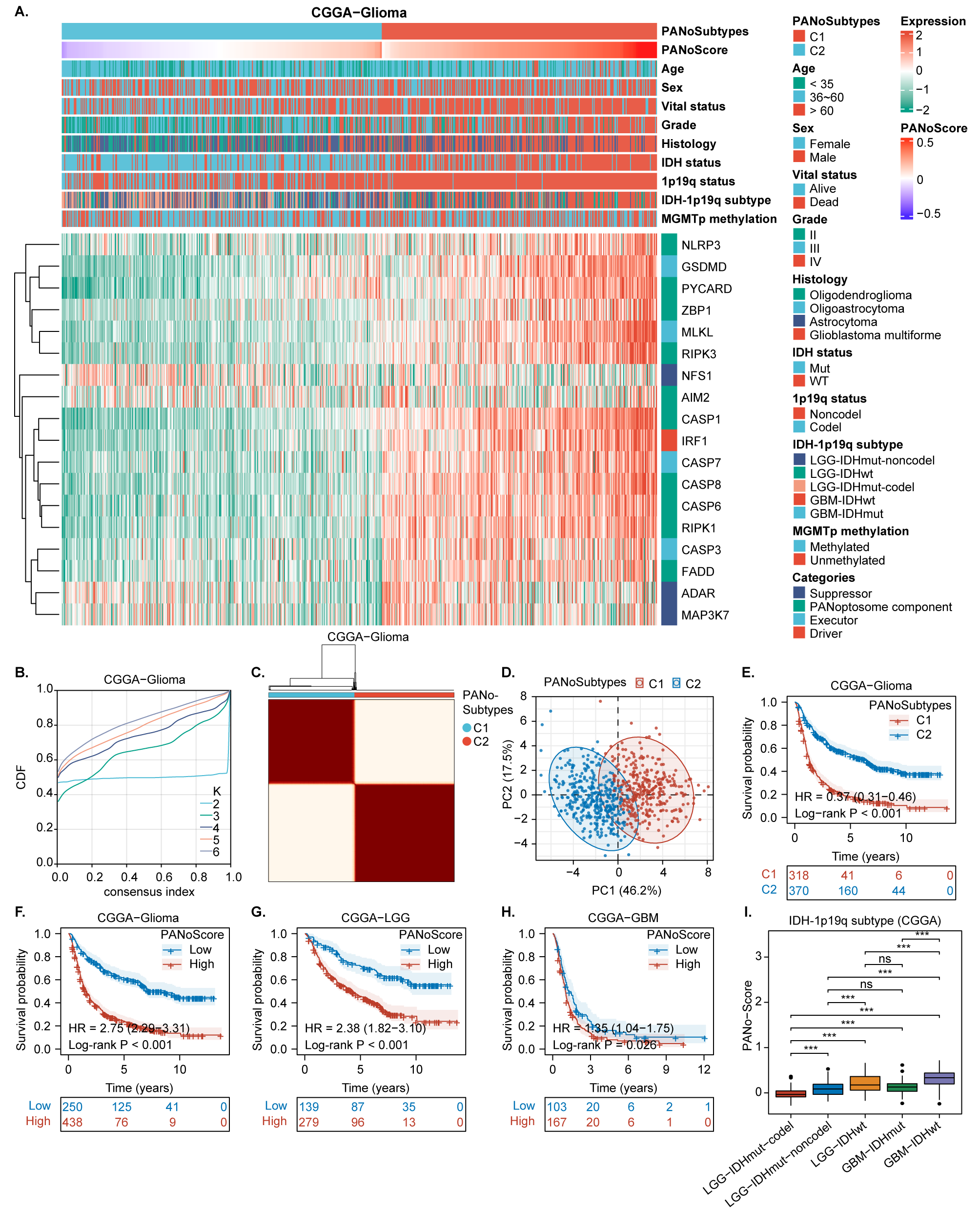

Supplement: Supplementary file 6 — Supplementary Material 6. [file 13046_2025_3301_MOESM6_ESM.tif]

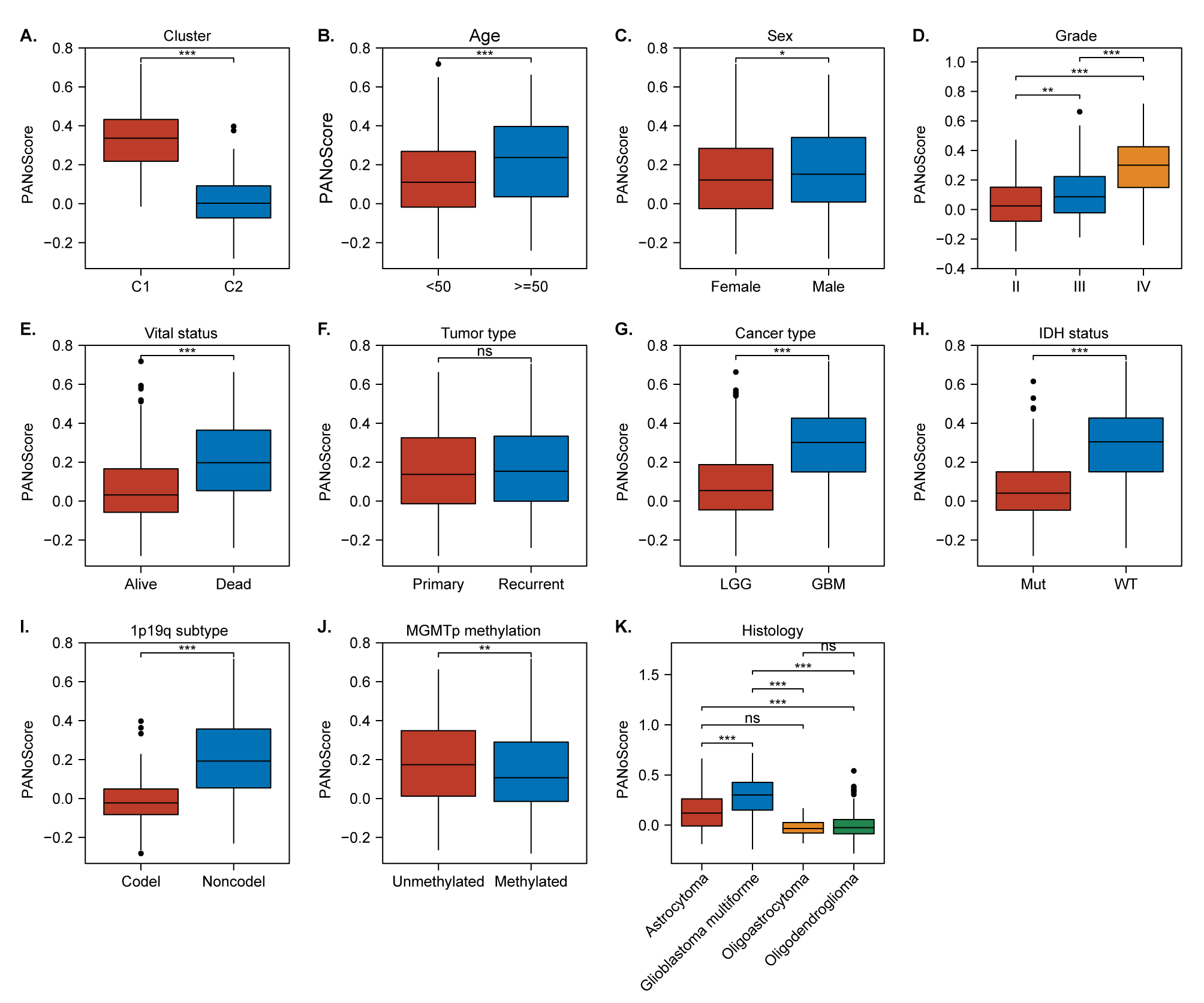

Supplement: Supplementary file 7 — Supplementary Material 7. [file 13046_2025_3301_MOESM7_ESM.tif]

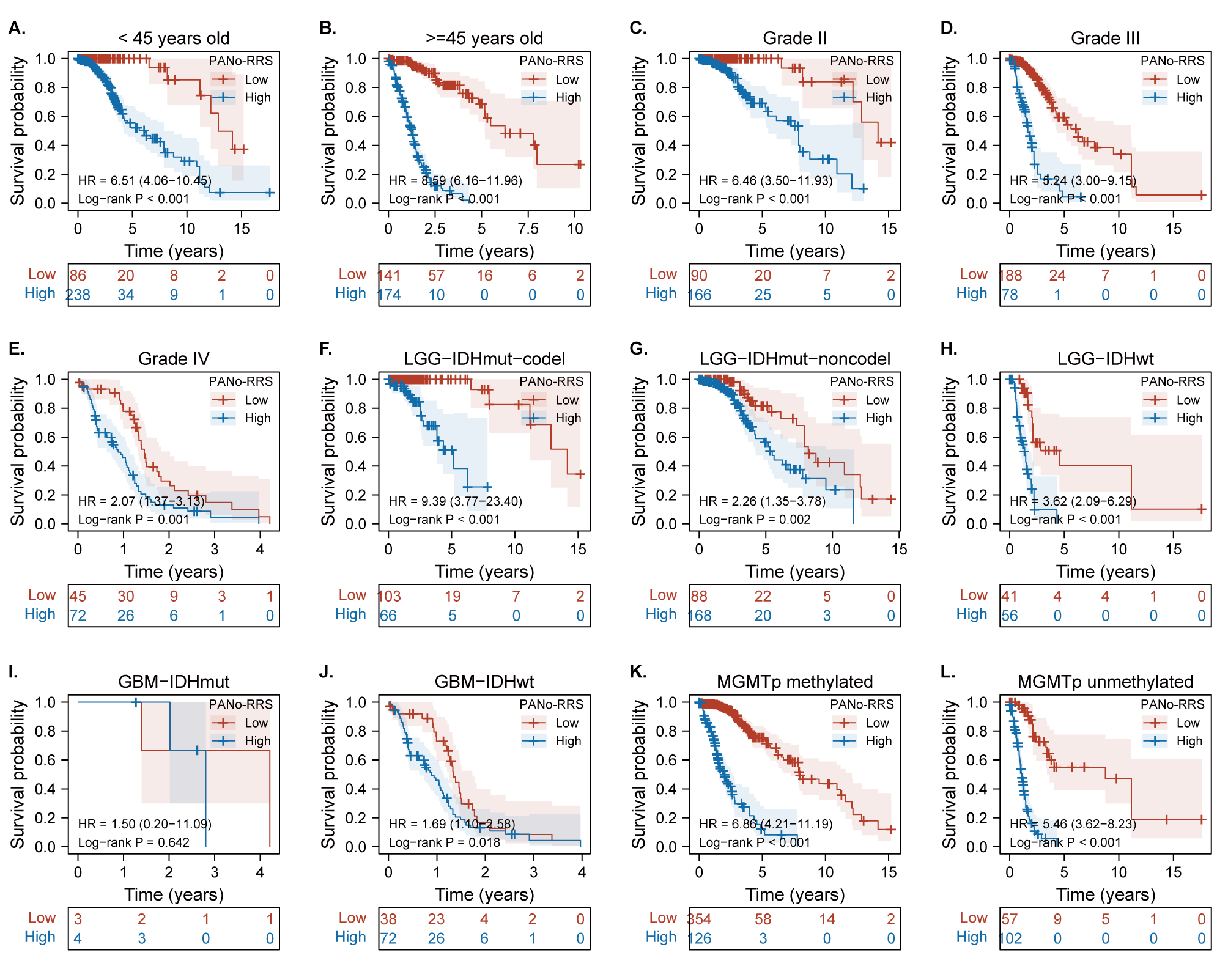

Supplement: Supplementary file 8 — Supplementary Material 8. [file 13046_2025_3301_MOESM8_ESM.tif]

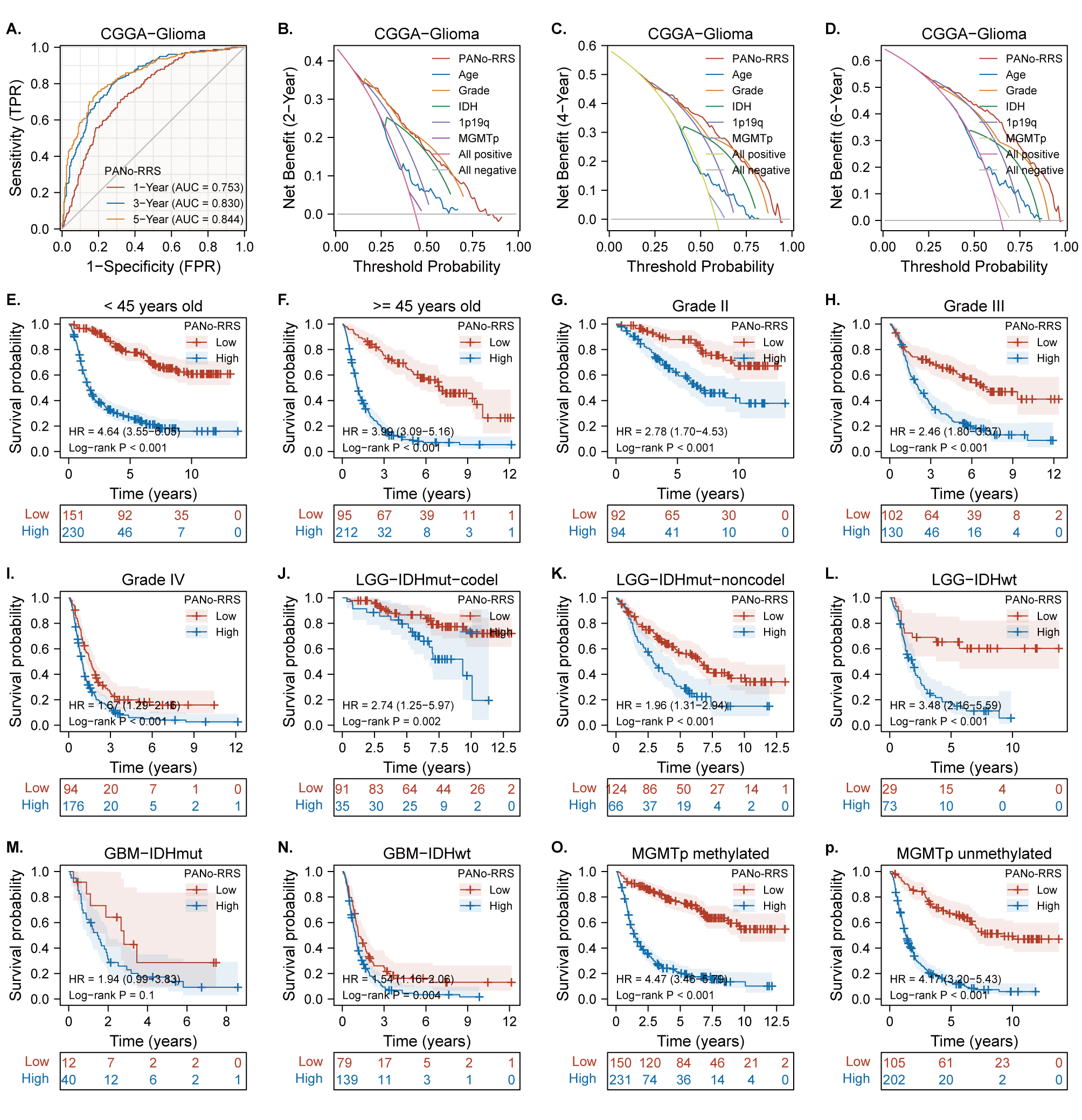

Supplement: Supplementary file 9 — Supplementary Material 9. [file 13046_2025_3301_MOESM9_ESM.tif]

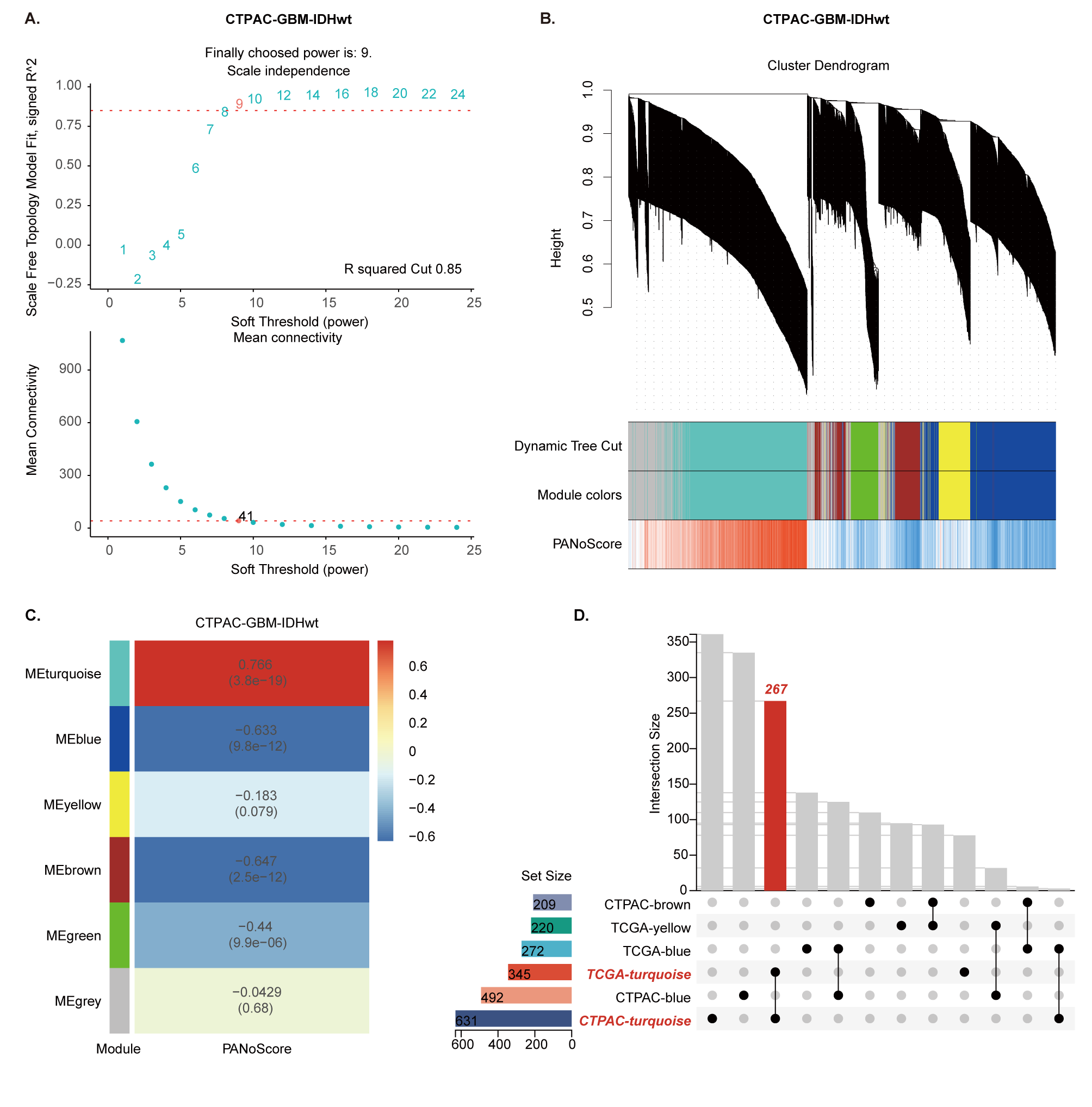

Supplement: Supplementary file 10 — Supplementary Material 10. [file 13046_2025_3301_MOESM10_ESM.tif]

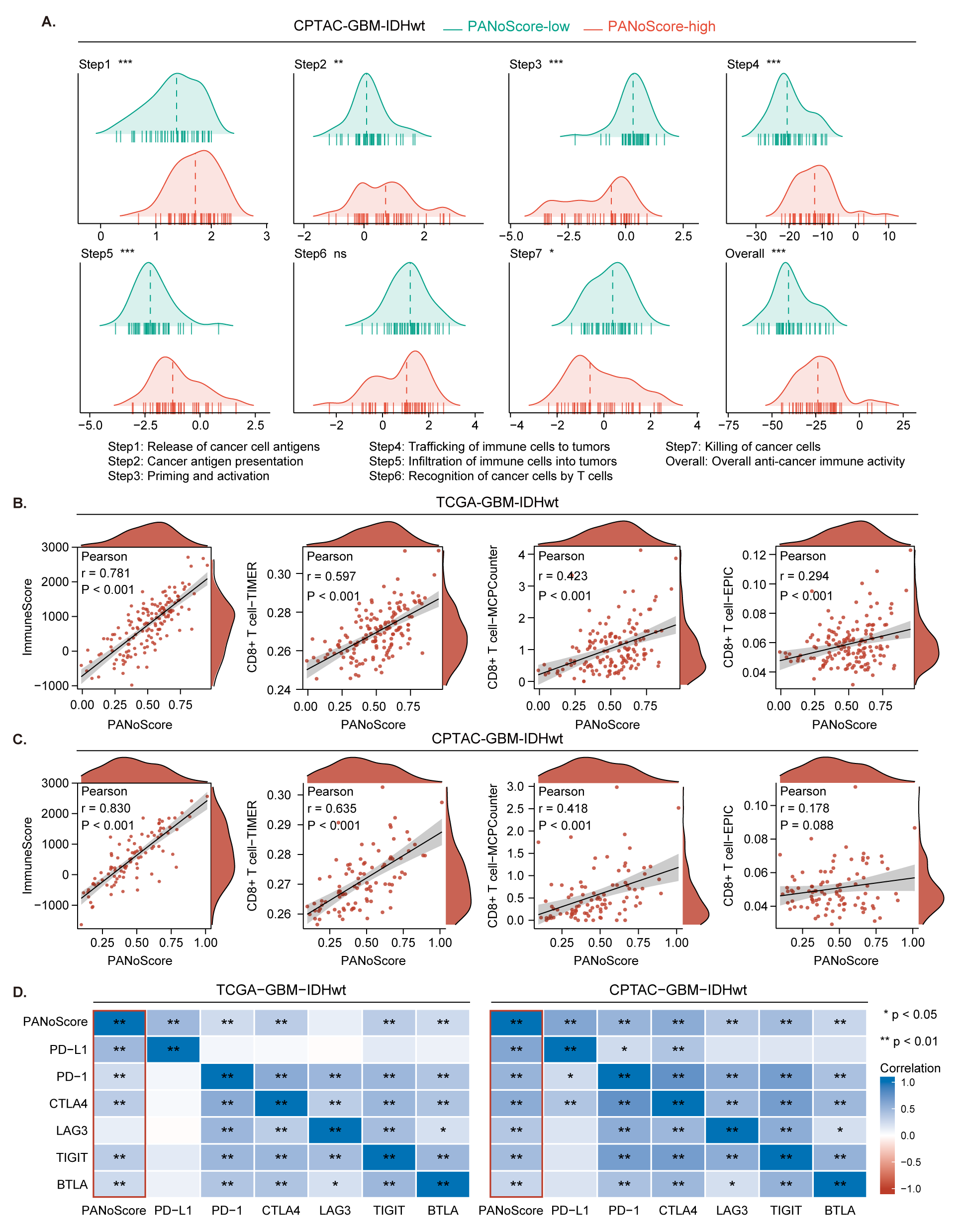

Supplement: Supplementary file 11 — Supplementary Material 11. [file 13046_2025_3301_MOESM11_ESM.tif]

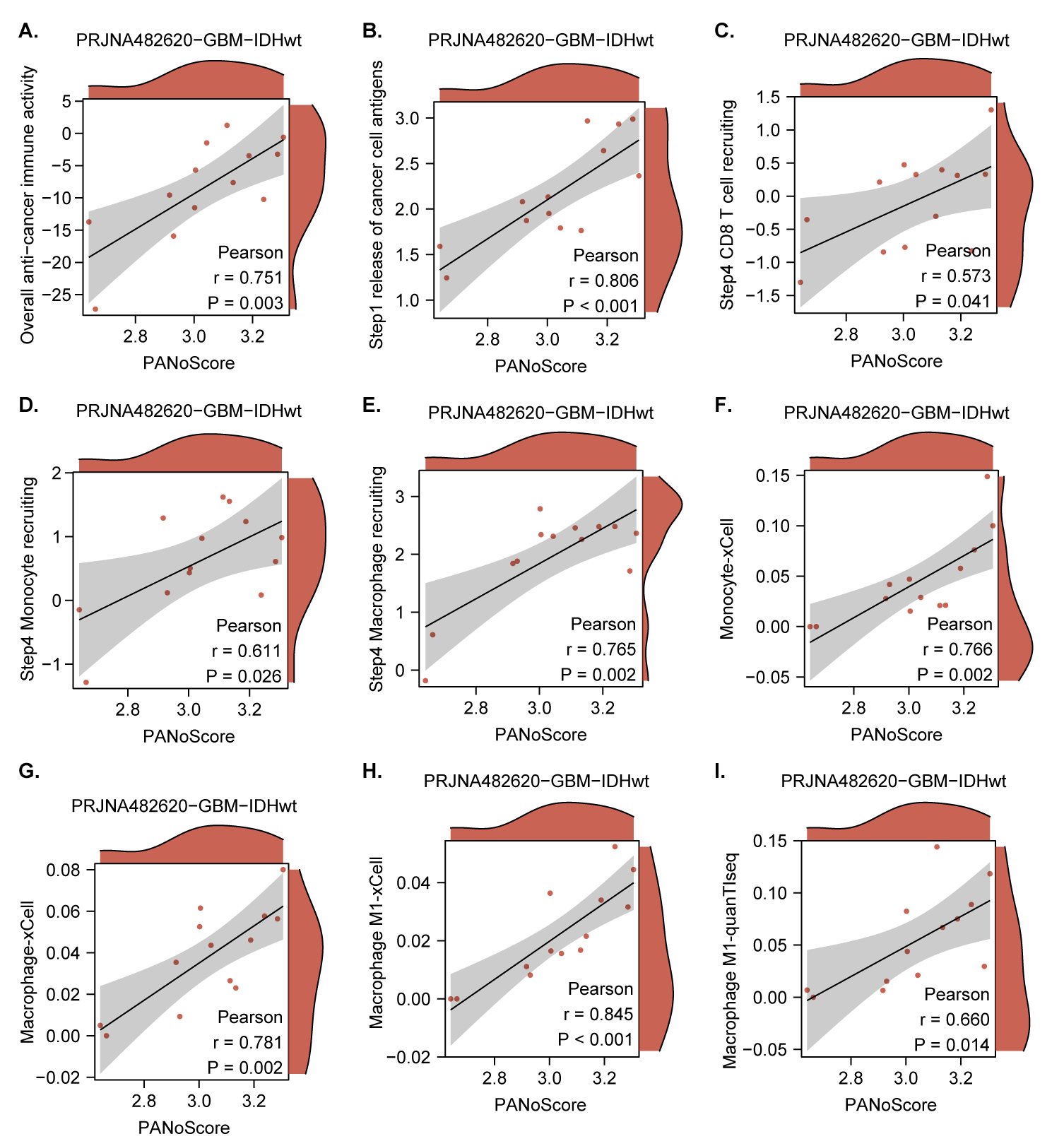

Supplement: Supplementary file 12 — Supplementary Material 12. [file 13046_2025_3301_MOESM12_ESM.tif]

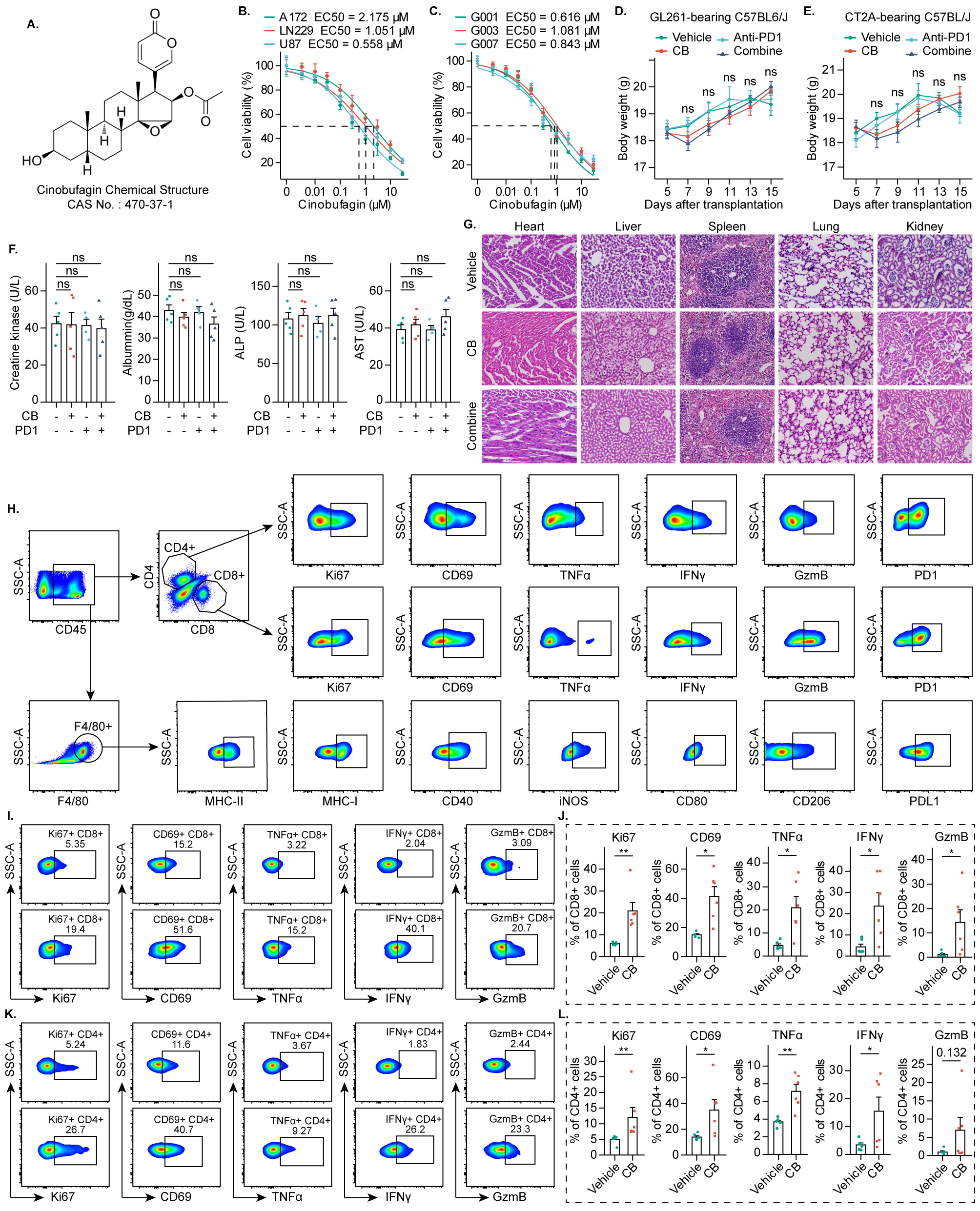

Supplement: Supplementary file 13 — Supplementary Material 13. [file 13046_2025_3301_MOESM13_ESM.tif]
